# A Mechanistic Model for Cooperative Behavior of Co-transcribing RNA Polymerases

**DOI:** 10.1371/journal.pcbi.1005069

**Published:** 2016-08-12

**Authors:** Tamra Heberling, Lisa Davis, Jakub Gedeon, Charles Morgan, Tomáš Gedeon

**Affiliations:** 1 Los Alamos National Laboratory, Los Alamos, New Mexico, United States of America; 2 Department of Mathematical Sciences, Montana State University, Bozeman, Montana, United States of America; 3 Computer Science Department, Montana State University, Bozeman, Montana, United States of America; Rutgers University, UNITED STATES

## Abstract

In fast-transcribing prokaryotic genes, such as an rrn gene in *Escherichia coli*, many RNA polymerases (RNAPs) transcribe the DNA simultaneously. Active elongation of RNAPs is often interrupted by pauses, which has been observed to cause RNAP traffic jams; yet some studies indicate that elongation seems to be faster in the presence of multiple RNAPs than elongation by a single RNAP. We propose that an interaction between RNAPs via the torque produced by RNAP motion on helically twisted DNA can explain this apparent paradox. We have incorporated the torque mechanism into a stochastic model and simulated transcription both with and without torque. Simulation results illustrate that the torque causes shorter pause durations and fewer collisions between polymerases. Our results suggest that the torsional interaction of RNAPs is an important mechanism in maintaining fast transcription times, and that transcription should be viewed as a cooperative group effort by multiple polymerases.

## Author Summary

Transcription of DNA by RNA polymerases is the first step of gene expression. It has been known that multiple RNA polymerases copying the same gene help each other to move faster, but the mechanism of this interaction is not known. We propose that the torque imposed by polymerase on helically twisted DNA and transmitted to the neighboring polymerases may play a central role in the observed cooperative behavior of polymerases. We incorporated the torque between polymerases into a basic stochastic elongation model and found that transcription times in this model match experimental data better than those of the same stochastic model without the torque effects. Using torque as the interacting mechanism of polymerases leads to significantly fewer collisions and traffic jams of polymerases. The resulting motion of polymerases resembles the motion of velocity-synchronized driverless cars on the highway.

## Introduction

Transcription is the first, and often the key, step in the control of gene expression. The process of transcription has several important phases. First, an RNA polymerase (RNAP) binds to a promotor sequence of a gene and initiates elongation. Next, the RNAP elongates down the DNA generating a single-stranded copy of RNA, and finally terminates, releasing the nascent copy of RNA. If the resulting RNA is mRNA, it is then translated by a ribosome to a chain of amino acids that fold to produce a protein. If the resulting RNA is rRNA or tRNA, it is not translated but provides a scaffold to facilitate binding of other proteins to form RNA-protein complexes such as ribosomes. In prokaryotes, both transcription and translation happen in the cytoplasm of a cell and can occur simultaneously. Therefore regulation of gene expression in bacteria, such as E. Coli, primarily happens at the transcriptional level [1, 2].

Elongation of RNAP along the DNA strand is not uniform, but is interrupted by frequent pauses. There are at least three different types of pauses; backtracking pauses, hairpin pauses, and ubiquitous pauses [3, 4]. Backtracking pauses and hairpin pauses have been shown to have a higher probability of occurring during transcription of specific sequences [5–7]. On the other hand, ubiquitous pauses are thought to have no dependence on DNA sequence and are equally likely to occur at any position along the DNA strand. It has been theorized that ubiquitous pauses are caused by a restructuring of the polymerase [4], but the exact cause remains an open question. These pauses are short (1–5 seconds) and occur approximately every 100 base pairs (bp) [4].

There has been substantial interest to understand the effect of the presence of pauses on the average *transcription time* and therefore output of the RNA, for highly transcribed genes. Presence of pauses may lead to traffic jams of RNAPs when one polymerase stops, affecting the trailing polymerases [8–10]. According to Klumpp *et. al.* [10], in their stochastic model RNAPs experienced a 40% reduction in the average elongation rate in dense traffic, amplifying the pause effect. This is similar to the results of a PDE model previously published by the authors [8, 9]. In less traffic, the RNAPs in the model experienced only a 12% reduction of the average elongation rate [10].

An ODE model was developed in 2008 that studied the interactions of simultaneously transcribing RNAPs [11]. In this paper, Tripathi et. al. were able to incorporate the mechanochemical cycles of each RNAP into their model using two main states; when the pyrophosphate PPi is bound to an RNAP and when PPi is not bound. Using a mean-field approximation, they calculated the average rate of RNA production. With highly transcribed genes, the interactions between RNAPs can have a large impact on elongation efficiency.

A prototypical example of a highly transcribed gene is an rrn operon in E. coli. Each E. coli genome has seven rrn operons whose transcription produces ribosomal RNA (rRNA) which provides a scaffold for a ribosome [12–14]. During conditions of rapid growth there are as many as 70,000 ribosomes in a cell. To keep up with high demand for ribosomes, 90% of transcription in fast growing E.coli produces rRNA and tRNA, and only 10% produces mRNA [15]. As a result, there is a high density of RNAPs on all rrn operons and a high transcription completion rate is imperative. Experimental measurements have shown that approximately 31% of an rrn operon is covered by RNAPs (about 51 RNAPs) [16] during high growth conditions, which strongly suggest that the polymerases interact either directly, or indirectly during transcription. This interaction appears to be cooperative. In vivo and in vitro experiments have demonstrated that presence of multiple RNAPs in close proximity can assist in increasing the average elongation rate. A trailing RNAP can help a paused RNAP to re-enter translocation, thereby decreasing the delay caused by pauses [17]. The magnitude of the cooperativity effect has not been firmly established. However, it is worth noting that the average elongation rate of RNAPs on the rrn operon is 90 nucleotides per second (nt/s) [10, 12, 18–20], which is about double the *in vivo* elongation velocity on protein coding genes [10, 21–23].

While the elegant paper of Epstein *et. al.* [17] firmly established the cooperativity effect, it did not propose a mechanistic explanation for this phenomena. In this paper we propose that the torsional force between the elongating RNAP and the DNA, caused by the helical structure of the DNA, may provide the mechanical underpinning for the interaction between elongating polymerases. The basis for our model is a set of experimental measurements by Ma and co-authors [24]. Using in vitro single-molecule experiments, they measured both the magnitude of torque exerted by elongating RNAP on DNA, and the effect of supercoiled DNA on RNAP velocity, pause density and pause duration.

We construct a model for transcription that substantially extends a basic stochastic model referred to as a Totally Asymmetric Simple Exclusion Process (TASEP), [2, 8, 10, 26–32]. In the most basic TASEP model each individual RNAP enzyme, hops along the DNA strand with a predetermined mean hopping rate provided that the forward site is unoccupied. The entire enzyme, spanning 35 nucleotides, translocates forward one nucleotide at a time as a unit, with the position of the RNAP being determined by the front of the enzyme. In addition to the basic TASEP, a mechanism for RNAP pausing can also be implemented. When pauses are included in the TASEP implementation, both the mean pause frequency and mean pause duration are constant and chosen a priori. In our model, Elongation with Torque Assisted Motion (ETAM), the rate of hopping depends on the torque between the polymerase and its closest two neighboring polymerases. The amount of torque is, in turn, the result of the relative motion of RNAPs on the DNA strand. Transcriptional pauses are included in the model as well. The mean hopping rate, mean pause frequency, and mean pause duration are dynamically updated within the model, and these parameters vary as the amount of torque varies for each RNAP. For any given RNAP that is translocating, the hopping rate and pause information depends upon the torque that is currently being experienced, and the subsequent motion is determined by sampling from the respective probability distribution functions. We base our model of the torque effects on experimental results by Ma [24] which experimentally measured the effect of torque on translocation (hopping) rate, pause duration and pause frequency. Over-twisting of DNA by shortening the distance between the polymerases increases (decreases) the translocation rate of the leading (trailing) polymerase, decreases (increases) the probability of entering a paused state for leading (trailing) polymerase and shortens (lengthens) the pause duration of the leading (trailing) polymerase.

ETAM simulation results show that the torque-based interaction between RNAPs results in a substantial cooperation effect between RNAPs. As a trailing RNAP approaches a leading RNAP, the resulting torque increases the effective elongation rate and reduces the likelihood and duration of pauses of the leading RNAP. At the same time, the effective elongation rate of the trailing polymerase decreases, while the likelihood and duration of pauses increases. As a result of this interaction, the duration of pauses decreases, and the average number of completed transcription events increases. The effect of this interaction is not unlike that of autonomously driven and communicating vehicles (“google” cars) on the road. By automatically adjusting velocity and helping each other to maintain proper spacing and shorten pauses, the collective motion of polymerases becomes more efficient with an average transcription time that is 37.5% shorter than that produced by the TASEP model. In this sense, the RNAPs are collaborating in order to transcribe the strand more efficiently than they would if they were traveling at a constant rate.

## Results

In order to examine the effect of the torque on the transcription simulation we compare several quantities of interest for both the ETAM and TASEP models. By comparing ETAM and TASEP, we isolate the effect of the torque mechanism. For clarity of the results presented in this section, we briefly describe the torque effects simulated in the ETAM model. The details of the torque computation and specific parameter values are found in the Methods section.

### Elongation with Torque Assisted Motion

DNA double helix structure makes one full rotation in approximately 10.5 base pairs [34]. RNAPs are large molecules that translocate along this twisted structure, which places constraints on the mutual motion of DNA and RNAP. If the DNA strand were fixed in space, an RNAP would have to rotate around DNA during translocation. The size of the RNAP and the packed environment inside the cell precludes this motion and a localized rotation of DNA has been observed [35, 36]. If the DNA strand were free to rotate, it could spin along its long axis as it enters a stationary RNAP. However, if DNA is fixed upstream of the elongating RNAP and the RNAP elongates without rotation, it applies torque to the DNA. This torque is stored within the portion of the DNA strand between the fixed end and the RNAP, and if the amount of torque is large enough, it can either preclude or facilitate the forward motion of itself and its neighboring RNAP.

The effect of torque on DNA—RNAP interaction was experimentally quantified in recent work of Ma et. al. [24] where a single-molecule optical trap experiment was employed in order to measure the effect of twist in a DNA strand on transcribing RNAP. In particular, they first applied a predetermined value of torque to a strand of DNA and then measured the elongation rate, the pause frequency and the pause duration of an RNAP elongating on the strand. Two types of twisting mechanisms are used to describe the applied torque. The first is over-twisting, and it is characterized by applying twist in a manner that shortens the length of the full rotation of the helix (measured in base pairs). Likewise, a twist that increases the length of the full rotation of the helix is termed under-twisting. Ma and collaborators observed that over-twisting decreases the elongation rate of the RNAP and increases both the likelihood and the duration of pauses. On the other hand, under-twisting was found to increase the elongation rate and to decrease both the likelihood and the duration of the pauses.

**Fig 1:**
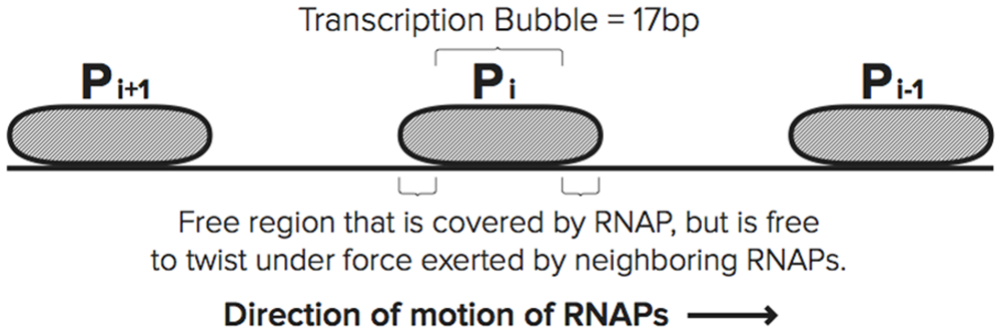
Polymerases *P*_*i* − 1_, *P*_*i*_, and *P*_*i*+1_ in order on the DNA strand. When *P*_*i*_ translocates, the DNA between *P*_*i* − 1_ and *P*_*i*_ will over-twist and the DNA between *P*_*i*_ and *P*_*i*+1_ will under-twist, increasing the elongation velocity of *P*_*i* − 1_ and *P*_*i*+1_.

To illustrate how these results can have an effect on the transcription of DNA by multiple polymerases, consider three consecutive polymerases on a DNA strand labeled as *P*_*i* − 1_, *P*_*i*_ and *P*_*i*+1_ in Fig 1. This notation is defined in detail in the section Incorporating Torque into Stochastic Model. We model the small segment of the DNA strand between two neighboring RNAPs as an elastic rod, and the elongation motion of one of the RNAPs imparts a twist within the elastic rod. The torque that results from this twisting motion is calculated using classical elasticity theory, and this calculation is detailed in the section Torque Between RNAP and DNA. A brief schematic overview of the motion of the RNAPs is presented below.

With respect to the motion of *P*_*i*_ along the strand, the RNAP represented by *P*_*i* − 1_ is referred to as the leading RNAP, and the RNAP labeled *P*_*i*+1_ is referred to as the trailing RNAP. If we assume that individual RNAPs never move at exactly the same time, when *P*_*i*_ moves forward, both *P*_*i* − 1_ and *P*_*i*+1_ provide anchors for the DNA strand. This movement imparts a torque to the portion of the DNA strand (one elastic rod) between *P*_*i* − 1_ and *P*_*i*_, as well as to the DNA strand between *P*_*i*_ and *P*_*i*+1_ (another elastic rod). The portion of the strand between *P*_*i* − 1_ and *P*_*i*_ will over-twist, and the portion of the strand between *P*_*i*_ and *P*_*i*+1_ will under-twist. Note that the over-twist will increase the elongation rate of *P*_*i* − 1_ (i.e. *P*_*i* − 1_ receives a “push from the back”) and under-twist will also increase elongation rate of *P*_*i*+1_ (i.e. *P*_*i*+1_ receives a “pull from the front”). It is noted in a later section that both of these effects tend to synchronize the motion of all three polymerases.

Our simulation results report the following quantities: the average transcription time, the average pause duration, the average collision duration time, the number of pauses and collisions, and the average transcriptional delay time experienced by an RNAP. Each of the above quantities is calculated over a range of initiation rates *α* ⋅ *β* where *β* is the average elongation rate of 90 nt/s and *α* ∈ [0.0001, 0.0115] using 11 discrete values within this interval. An *α* value of 0.0001 corresponds to an initiation every 111 seconds on average, while *α* = 0.0115 would have an initiation every 0.96 seconds on average. For each value of *α*, we performed 50 simulations of both ETAM and TASEP and ran the simulation for 10,000 simulated seconds. This ensures a sufficient amount of data is collected for accurate results when compiled and averaged together. In each of the fifty simulations we record the start and end time of each RNAP transcription process, and we also record both the beginning and the end time of each pause and collision.

### Average Transcription Time

**Fig 2:**
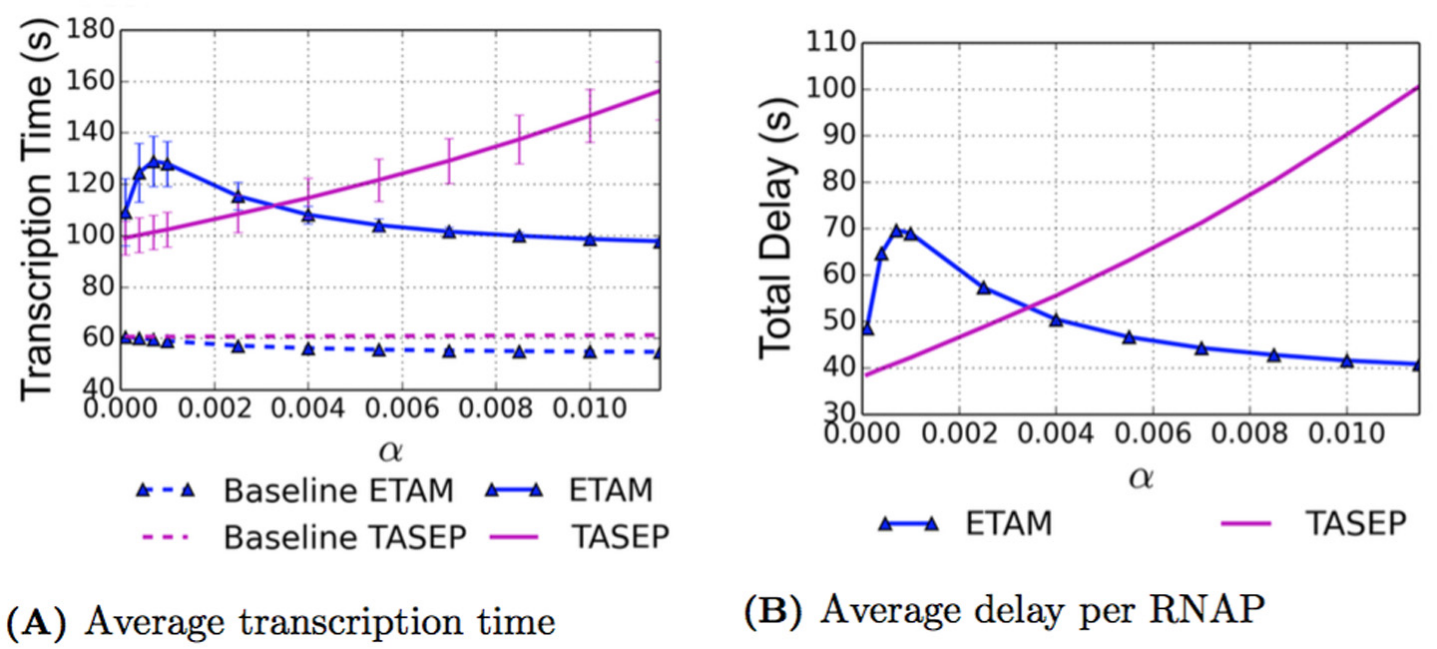
As a function of the initiation rate *α* ⋅ *β*, we present the average transcription time (A) and the average total delay per RNAP (B). The ETAM model (blue triangles) and TASEP model (magenta) are both plotted for comparison. The dashed lines represent baseline simulations with no pauses. The error bars for the transcription time represent the standard deviation, with the standard deviation on the ETAM model decreasing as *α* increases.

Experimental results from physicists and biologists give an average transcription time for the rrn gene of approximately 60 seconds [4, 16], and in [16], the authors also assert that the rrn gene is, on average, approximately 31% covered. This corresponds to an average velocity of 90 nt/s. With the physical parameters used for both the TASEP and ETAM model simulations, we attempt to mimic the biological case of transcription of this gene. In order to obtain the average transcription time per RNAP, the transcription time for each RNAP within the simulation is recorded, and these values are averaged over all of the RNAPs for each specific initiation rate. The results can be seen in Fig 2A where data is presented for two situations. In addition to the simulations for both TASEP and ETAM models with pauses, we also performed numerical simulations for both models in the case of no pauses for comparison purposes. We refer to the case without pauses as the “baseline” case for each model. While the RNAPs could still experience collisions for the baseline case, the number of collisions was significantly lower, see the curves with the dotted lines in Fig 2A. This baseline model allows us to calculate the transcription time for an RNAP without any transcriptional delays caused by pauses, and we note that for both models, the average transcription time is close to 60 seconds and agrees well with numbers reported in the literature. For *α* = 0.0115, we observe a 61.23 second average transcription time for the baseline TASEP model and a slightly faster average transcription time of 54.7 seconds for the baseline ETAM model.

Examining the case where transcriptional pauses are introduced into each of the models, we see very different effects, and these are shown in the solid curves of Fig 2A. For *α* = 0.0115, the average transcription time for TASEP is approximately 156.25 seconds, which for a DNA strand of 5450 nucleotides corresponds to an average velocity of 34.88 nt/s. This rate is significantly lower than the 90 nt/s resulting from an experimental average transcription time of 60 seconds for the rrn gene reported in [4, 16]. In contrast, for the ETAM model at *α* = 0.0115, the average transcription time was approximately 97.73 seconds, which corresponds to an average velocity of 55.77 nt/s. This average velocity agrees much better with the experimental values. The significant difference in average transcription times between the two models is attributable to differences in the average number of pauses and their durations, as well as differences in the average number of collisions between RNAP’s in the two models. In the following sections we carefully examine the effect of torque on these quantities.

### Average Number and Average Duration of Pauses

**Fig 3:**
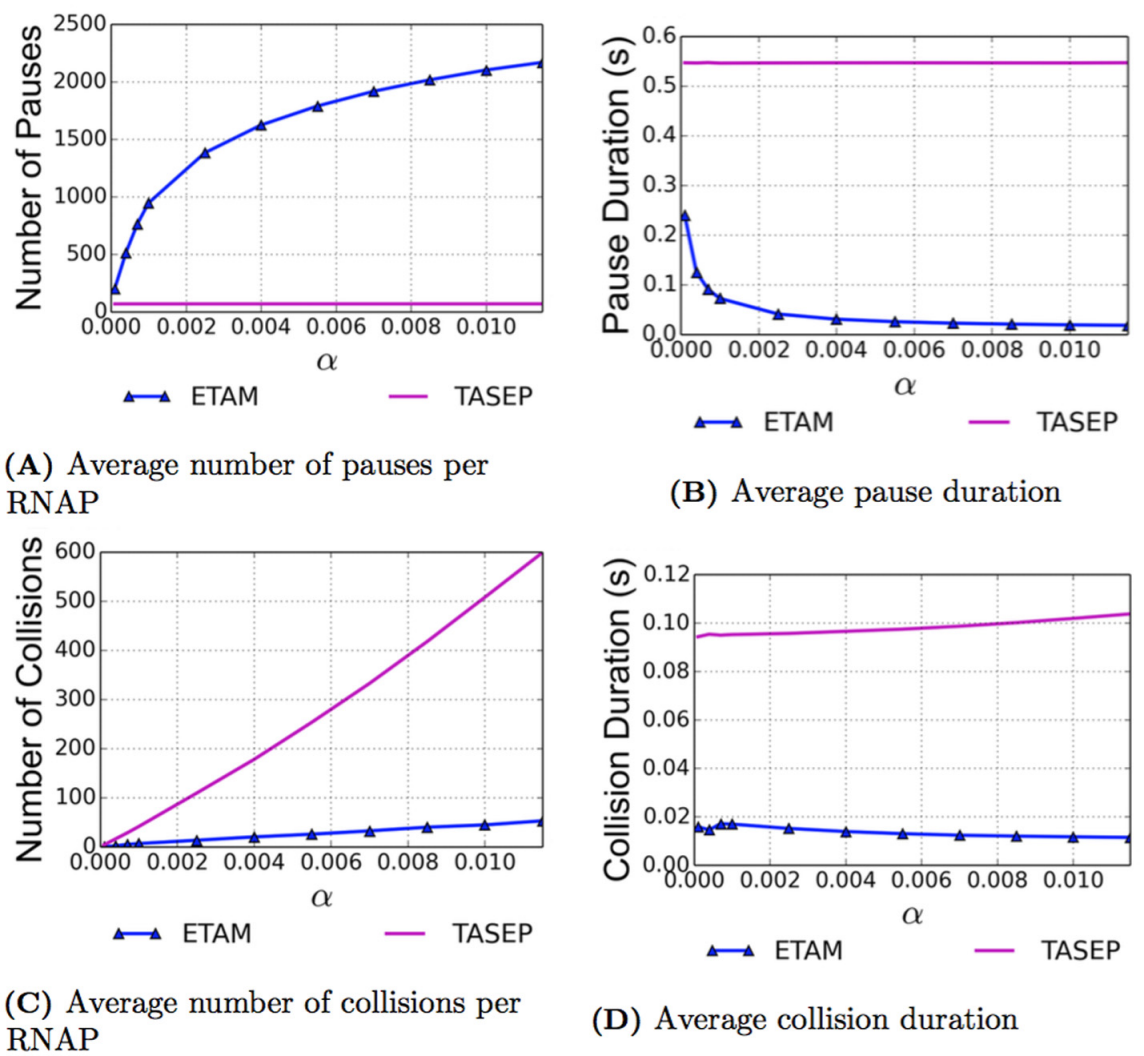
As a function of increasing initiation rates determined by *α*, the pause and collision results are presented for the ETAM model (blue triangles) and the TASEP model (magenta). The number of pauses and collisions are computed as the average number per RNAP. The average number of pauses per RNAP is presented in (A) with the average pause duration in (B). Similarly the average number of collision per RNAP is given in (C) with the average collision duration in (D). RNAPs in the ETAM model experience significantly fewer collisions and shorter pause durations than their TASEP counterparts.

In the TASEP model simulations, each RNAP experienced, on average, 70 pauses with an average duration of 0.55 seconds. As expected, these results are constant for the TASEP model over the entire range of initiation rates as seen for the magenta curves in Fig 3A and 3B respectively. For the ETAM model, the average number of pauses experienced by an RNAP varies significantly over the range of initiation rates included in the simulations. The number of pauses increases monotonically from 202.69 for *α* = 0.0001 to 2169.32 for *α* = 0.0115. This is a 970.3% increase over the range of initiation rates; moreover, for the entire collection of these simulations, the average number of pauses was consistently larger than that of the TASEP model, see Fig 3A. For the largest value of *α*, RNAPs in ETAM experienced 2999% more pauses than RNAPs in TASEP.

This result can be intuitively explained based on the construction of the ETAM model discussed in the Methods Section. If there are more RNAPs on a DNA strand, the RNAPs will be more likely to interact and the interaction they experience will likely be stronger than if there were fewer RNAPs. This is because the distance between RNAPs is smaller, on average, for a strand with a higher percentage of RNAP coverage. Therefore one would expect RNAPs to experience more pauses in an environment with more coverage (higher values of *α*) than they would in an environment with less coverage (lower values of *α*).

An important and interesting observation that accompanies the preceding results is the data for the average duration time of these transcriptional pauses. Our results indicate that in the ETAM model, the average duration time of the pauses decreases significantly for higher initiation rates, see Fig 3B. The duration time decreased 91.7% over the range of initiation rates, from 0.24 seconds when *α* = 0.0001 to 0.02 seconds when *α* = 0.0115. Note that the values of pause duration on the order of 0.02 seconds would not be detectable in experiments, and so very likely the motion of polymerases at these coverages will experience fewer observable pauses. At the highest value for *α*, the average pause duration time is 96.4% lower in ETAM than in TASEP. We propose that the effects of the torque mechanism on the average duration time of pauses can be summarized as follows. While an RNAP is more likely to pause in regimes with higher initiation rates, the effects of torque, that is, the “push from the back” and the “pull from the front,” are stronger when the neighboring elongating RNAPs are closer to the paused RNAP. Therefore, the paused RNAP can be pushed or pulled “out” of its pause state and into active elongation by means of these torsional effects much more quickly than in a regime where the pause duration time is determined by purely stochastic effects.

### Number and Duration of Collisions

**Fig 4:**
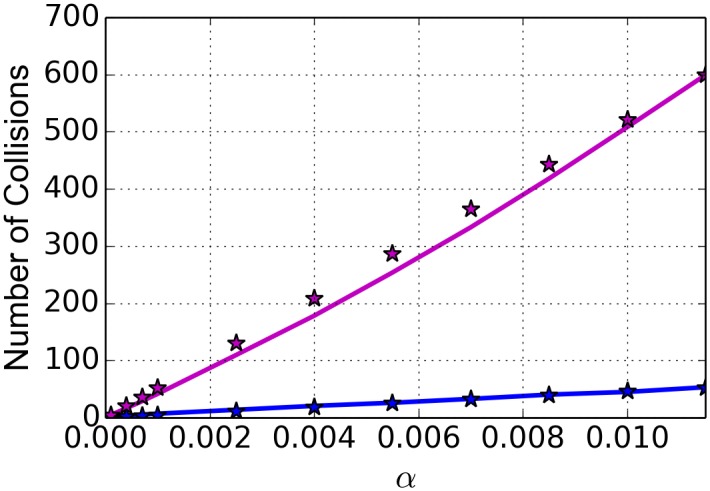
Linear fit for the average number of collisions experienced per RNAP. The collisions in the results for TASEP (magenta) and compared to the linear fit (magenta stars) and similarly for the ETAM results (blue and blue stars).

We hypothesize that we can explain results of Fig 3C by the fact that the torque mechanism of the ETAM model allows transcribing RNAPs to maintain their spacing relative to their neighbors and to decrease the number of collisions (as described in the Methods Section under Incorporation of Collisions) that occur among polymerases. In order to investigate this hypothesis, we monitor and record the average number of collisions that occur and the time durations of those collisions for both the TASEP and the ETAM models. The initial intuitive expectation is that one would observe an increase in the number of collisions as the initiation rates increase (or as the percent of coverage increases). That is, for a larger coverage of RNAPs on the DNA strand, collisions should become more likely. Likewise, if there are very few RNAPs on the DNA simultaneously, collisions are unlikely. As shown in Fig 3C, the data for the average number of collisions behaves as expected. However, the number of collisions increases much more rapidly in the case of the TASEP model than for the ETAM model. For the highest initiation rate simulated, RNAPs in the TASEP model experienced 1026.5% more collisions than RNAPs in the ETAM model. In particular, results show that approximately 550 more collisions per RNAP occur within TASEP; we observe an average of 53.18 collisions per RNAP in the ETAM model and an average of 599.09 collisions per RNAP in the TASEP model. For each of the models, the data sets shown in Fig 3C were fit with a linear least squares model, and the average number of collisions experienced by an RNAP has approximately linear growth over the range of *α* values for both cases. The linear fits can be seen graphically in Fig 4, and the equations for the lines *C*_*τ*_(*α*) for ETAM and *C*(*α*) for TASEP are given by

$$\begin{array}{ccc} C_{\tau }\left(\alpha \right) & = & 4609.65\alpha \\ C(\alpha ) & = & 52175.4\alpha \end{array}$$

Computing a ratio of the slopes of these two lines, we observe that the number of collisions for the ETAM results is growing at approximately 9% of the rate of the number of collisions of the TASEP results.

Given that RNAPs in the TASEP model collide much more often, the average duration time of each collision becomes critical. Fig 3D shows that for the largest initiation rate, *α* = 0.0115, the average duration time of a collision is significantly longer in TASEP, 0.104 seconds, than in ETAM, 0.011 seconds. Fig 3D indicates that RNAPs in the TASEP model tend to experience collisions that last approximately five times as long as those in the ETAM model. Moreover, this effect is consistent over the entire range of initiation rates included in the study. We believe this indicates that the torque is contributing to the RNAP’s ability to maintain a degree of spacing and distance with its neighbors, thereby reducing the number of collisions that occur for the ETAM case. In addition, when collisions occur in the ETAM model, they tend to be very short in duration.

In summary, the inclusion of torque effects into the basic stochastic model seems to allow transcribing RNAPs to dynamically manage elongation velocity and spacing so as to avoid collisions and traffic jams. While the RNAPs experienced more pauses in the ETAM simulations, these pauses were significantly shorter in duration than those of the TASEP simulations, with far fewer collisions and shorter collision durations. To investigate more closely the effect of torque on pauses, collisions and their duration we attempt to summarize these effects by computing average transcriptional delay experienced by a polymerase in each model.

### Average Transcriptional Delay

During the transcription process, an RNAP experiences both pauses and collisions, each with a certain time duration. These interruptions cause active elongation of the RNAP to cease until the RNAP is able to move again. The amount of time that an RNAP is unable to translocate contributes to the delay that the RNAP experiences. To quantify this concept, we define the average total delay to be sum of both the average delay due to pauses and the average delay due to collisions. The average total delay per RNAP is computed with the formula

$$\begin{array}{ccc} {\text{Delay}}_{\text{total}} & = & {\text{Delay}}_{\text{pause}}+{\text{Delay}}_{\text{collision}}\\ & = & \left(\text{ave}\#\text{ of pauses per RNAP})\;\cdot \;(\text{ave pause duration}\right)\\ & + & \left(\text{ave}\#\text{ of collisions per RNAP})\;\cdot \;(\text{ave collision duration}\right) \end{array}$$

the results of which can be seen in Fig 2B. Specific values of the total delay over the range of initiation rates for each of the ETAM and TASEP models can be found in Tables 1 and 2, respectively. The average delay experienced by an RNAP in the TASEP model increased from 38.52 seconds when *α* = 0.0001, to 100.61 seconds when *α* = 0.0115. Conversely for the ETAM model, the average delay decreased from 48.63 seconds to 40.82 seconds over the same range of initiation rates.

**Table 1:** Results for ETAM model: percent of the strand covered by polymerases, average transcription time (s) per RNAP, average collision delay (s) per RNAP, average pause delay (s) per RNAP, and average total delay (s) per RNAP.

*α*	% Coverage	Time	Collision Delay	Pause Delay	Total Delay
0.0001	1.26	109.12	0.01	48.62	48.63
0.0004	3.42	124.44	0.03	64.70	64.73
0.0007	5.51	128.88	0.09	69.59	69.68
0.001	7.52	127.91	0.12	68.89	69.01
0.0025	14.88	115.36	0.21	57.12	57.33
0.004	20.55	108.04	0.29	50.17	50.46
0.0055	25.42	103.99	0.34	46.33	46.67
0.007	29.61	101.47	0.41	43.90	44.31
0.0085	33.31	99.85	0.48	42.31	42.79
0.01	36.81	98.59	0.53	41.08	41.61
0.0115	39.70	97.73	0.61	40.20	40.81

**Table 2:** Results for TASEP model: Percent of the strand covered by polymerases, average transcription time (s) per RNAP, average collision delay (s) per RNAP, average pause delay (s) per RNAP, and average total delay (s) per RNAP.

*α*	% Coverage	Time	Collision Delay	Pause Delay	Total Delay
0.0001	1.21	99.02	0.40	38.12	38.52
0.0004	2.90	100.15	1.57	38.26	39.83
0.0007	4.55	101.23	2.71	38.30	41.01
0.001	6.20	102.27	3.95	38.26	42.21
0.0025	14.26	108.33	10.45	38.33	48.78
0.004	21.75	114.56	17.20	38.34	55.54
0.0055	29.05	121.54	24.72	38.41	63.13
0.007	36.08	129.00	32.82	38.41	71.23
0.0085	42.89	137.35	41.85	38.41	80.26
0.01	49.40	146.55	51.75	38.43	90.18
0.0115	55.26	156.25	62.14	38.47	100.61

For the ETAM model, the decrease in delay values for increasing initiation rates (and thus increasing coverage), is evidence to suggest that the torque contributes to an increase in efficiency with multiple RNAPs transcribing simultaneously. Moreover, the comparison of these two models allows us to observe that, in the TASEP model, the phenomena that drives the total delay is that of the delays due to collisions (as opposed to the delays due to transcriptional pauses experienced). Table 2 clearly demonstrates that, for the TASEP model, the delay which the RNAPs experience due to pauses remains virtually constant over the entire range of initiation parameters and that the fact that the total delay is increasing, is almost exclusively attributable to the delays due to collisions. In contrast, Fig 3C and Table 1 demonstrate that the torque mechanism included in the ETAM model prevents many collisions from happening, and it also decreases the amount of delay that RNAPs incur from those few collisions that actually do occur. This is especially apparent with the higher initiation rates. The torsional interaction between RNAPs leads to far more efficient transcription, especially in the case of high coverage of the DNA strand.

The torque provides a mechanism for the RNAPs to interact and to cooperatively prevent collisions from occurring. If an RNAP pauses, the trailing RNAP will slow down and/or enter a pause with a much higher probability due to the increasing torque applied to it as its elongation continues. The trailing RNAP will likely enter a pause or it may push the leading RNAP into active elongation before a collision occurs. The evidence of this interaction can be seen in the difference in the number of collisions experienced per RNAP in the two models in Fig 3C and Tables 1 and 2.

The comparison of the ETAM and TASEP models leads us to propose that torque is an important mechanism in the regulation of transcription. Neighboring RNAPs may interact with each other using torque to optimize their elongation efficiency as a group effort as opposed to an individual process as suggested in [17]. In this paper, Epshtein et. al. show that transcription times are faster with multiple RNAPs present on the strand as opposed to the case of a single molecule transcribing. The ETAM simulations for *α* = 0.0001 have an average transcription time per RNAP of 109 seconds, with an average time between initiations of 111 seconds, see Table 1. With this parameter setting, the simulation is essentially a model of transcription by a single polymerase. The average transcription time for *α* = 0.0115 decreased nearly 12 seconds from the lower value of *α*. This is largely due to the paused RNAPs being pushed back into active elongation by their neighboring polymerases, a phenomenon suggested by Epshtein and Nudler [17]. Thus far, our results are being presented in terms of the average values, we now consider the variance of the average transcription time, the average pause duration, and the average collision duration; these are the quantities that have received careful consideration for model comparison.

### Variance of Transcription Time, Pause Duration and Collision Duration

**Fig 5:**
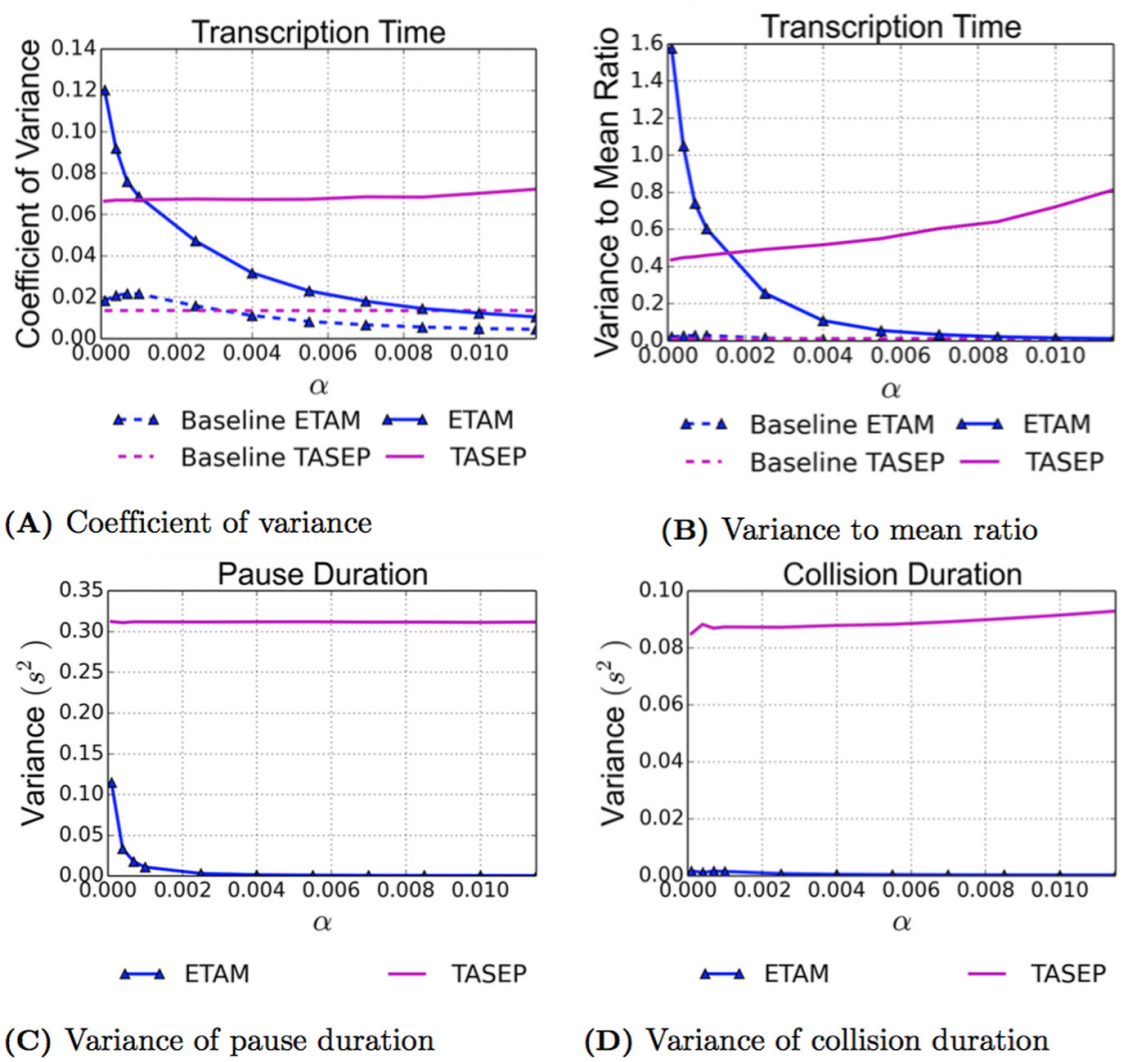
As a function of increasing initiation rates determined by *α*, the coefficient of variation (A), and the variance to mean ratio (B), are given for the transcription time. Both ETAM and TASEP models are presented as well as their baseline results (no pauses). The variance of the pause duration (C), and collision duration (D), are presented for the ETAM model (blue triangles) and the TASEP model (magenta).

The coefficient of variation and the variance to mean ratio are presented in Fig 5(A) and 5(B) respectively. In both of these variance measures, the variability from the mean of the ETAM model decreases significantly and becomes very small in as the initiation rates increase while the TASEP model variability continues to grow. The baseline models, simulated with no pauses, are included in order to show that the variance is very close to zero for both models in the absence of pauses. With the addition of pauses, the possibility of prolonged traffic jams and collisions immediately increases the variance in both models. For the case of ETAM, as more RNAPs are added to the DNA strand, the torque interaction between polymerases drives the variance in transcription time with respect to the mean down towards zero again, as shown by the coefficient of variation and the variance to mean ratio.

For both the pause duration and the collision duration, the variance for ETAM decreases as *α* increases. Therefore when there are fewer RNAPs on a DNA strand, the higher variance in pause duration indicates that the pause duration for the RNAPs is somewhat variable. However, as more RNAPs are transcribing simultaneously, the case for the higher *α* values, this variance goes to zero for both the pause duration and collision duration. The torque interaction between RNAPs is much stronger when RNAPs are closer together. This allows the RNAPs to communicate, and it stabilizes the elongation process. As a result of the decrease in pause durations and number of collisions, the RNAPs do not experience large traffic jams that can cause a great deal of variability in transcription time. In higher densities, the RNAPs can also push a paused RNAP back into elongation which causes uniformly short pause durations with very little variability.

The variance for the TASEP model is significantly different than for ETAM. Since the mean hopping rate, mean pause frequency as well as the mean pause duration for TASEP are all prescribed in the model, independent of the parameter *α* and constant throughout the simulation process, it is expected that the variance for the pause duration remains constant over the range of initiation rates in the case of TASEP. However, the variance for the collision duration slightly increases, and the variance measures for the transcription time increase considerably. Although the variance for the collision duration only slightly increases, the average number of collisions increases significantly as *α* increases, see Fig 3C, and this results in the increased variability of the average transcription time at higher values of *α*. Without torque, the RNAPs in the TASEP model are unable to work together. Therefore, when there are many RNAPs elongating on the same DNA strand, an individual RNAP is more likely to experience a traffic jam. The increased frequency with which these traffic jams occur and the variability of the length of these traffic jams can cause a large difference in transcription times for individual RNAPs.

## Discussion

By incorporating the torque mechanism into a basic TASEP model of transcription we are able to see a cooperative effect among transcribing RNAPs. This effect was noted experimentally in 2003 by Epshtein and Nudler [17]. At the time, the mechanism causing this behavior was unclear. After the recent developments by Ma et al [24], and results from our model simulations, we propose that the torsion on the DNA caused by RNAP transcription is allowing the RNAPs to communicate with each other in order to maintain proper spacing, thereby avoiding collisions, and to increase the rate of transcription. A theoretical examination of the effect of the torque mechanism proposed here can be found in the Methods Section under Mean Field Approximation Model. A classical car following model that includes forces from both the leading and trailing RNAPs yields a steady state density-velocity relationship that qualitatively explains why the torque mechanism generates cooperative behavior among the neighboring RNAPs.

The cooperation between RNAPs is clearly seen from the results of our stochastic model, ETAM, which incorporates torque into a basic TASEP model. We compare the results of this model with those of the TASEP model to isolate the effect of the torque. There are two results that clearly demonstrate this cooperative effect: the average number of collisions each RNAP experiences and the average pause duration.

With a high initiation rate of RNAPs onto the DNA, each polymerase experiences on average 550 fewer collisions with neighboring RNAP during the course of transcription when the elongation is regulated by torque. This is a direct result of the torque allowing the RNAPs to communicate with the polymerases that are closest to them. During a simulation of TASEP, an RNAP will elongate until it either pauses or is stopped because the next nucleotide downstream is occupied by a paused polymerase. With ETAM, as an RNAP elongates close to a paused RNAP, the resisting torque experienced by the elongating RNAP makes it much more likely to pause. At the same time, the paused RNAP experiences an assisting torque from the elongating RNAP which can push it out of a pause and into active elongation. This interaction prevents an average of 550 extra collisions from occurring in high coverage regime.

The average pause duration is the other quantity most affected by the torque. With ETAM the pause duration is not a fixed quantity but can be dynamically recalculated to account for the actions of neighboring RNAPs. Pause durations can be shortened when the polymerases surrounding the paused RNAP elongate. Simulation of ETAM produced, on average, 0.02 second pauses as opposed to 0.55 second pauses simulated in TASEP. When comparing the average transcription time per polymerase we see an even more striking difference. The ETAM model shows polymerases experiencing an average transcription time of 97.73 seconds as opposed to 156.25 seconds in TASEP. The delay a polymerase experiences as a result of collisions and pauses has the largest effect on the overall transcription time. With fewer collisions and shorter pause durations, the RNAPs simulated in ETAM have significantly less delay resulting in far more efficient transcription.

As promising as these results are, they depend on how we fit limited data for velocity, pause frequency, and pause duration into functions of torque as discussed in the Methods Section under Incorporating Experimental Data to Determine the Effect of Torque. With a small amount of data points available and no data for values near the stall torque and melting torque, the models that we use to fit these functions near those end points are somewhat arbitrary. These high and low values for torque are calculated quite often with a high density of RNAPs on a DNA strand since the torsional interactions are so strong. As a result, the performance of the ETAM model depends on the choice for these functional descriptions when the torque values are sampled from regions where no experimental data is currently available. This issue is explored within the Methods Section under Results from Different Pause Frequency Functions.

As nanotechnology continues to improve, our hope is that data will become available for velocity, pause duration, and pause frequency at both very low and very high torque values. This will allow us to better fit our model to the data without needing to make assumptions for the extreme cases. Even with the limited data, the cooperation effect is evident in the ETAM results, with shorter transcription times in the simulations for the range of high initiation rates. With more polymerases transcribing DNA simultaneously, each RNAP experiences less delay than RNAPs transcribing with a smaller amount of polymerases. In the case of the highly transcribed genes, transcription can be viewed as a group effort, with torsional interactions allowing all of the RNAPs to transcribe more efficiently.

## Methods

We construct the ETAM (Elongation with Torque Assisted Motion) model by extending the construction of a basic TASEP framework. The ETAM construction is discussed in detail, and then we briefly describe the simplifications to ETAM which result in the original TASEP model. We begin by discussing the fundamental aspects of TASEP that provide the foundation of ETAM.

### TASEP Model

TASEP is a stochastic model that has been used to describe the process of both transcription and translation [2, 8, 10, 26–32]. In TASEP, each RNAP enzyme hops forward with a constant rate *β* on a 1-dimensional strand with a discrete and finite number of sites. RNAPs cannot occupy the same site and therefore will cease active elongation if the next site is occupied (we will refer to this as a collision). Only when the next site is vacated will elongation resume. We have implemented open boundary conditions where the RNAP enzymes enter the strand at a given rate and exit the strand once they reach the opposite end. Specifically, each RNAP enters our simulation (initiates) with a rate of (*α* ⋅ *β*), and leaves the simulation (terminates) with a rate of (*γ* ⋅ *β*). This is consistent with a differential equation model for transcription proposed originally in the late 1960s [33]. Therefore *α* and *γ* are scalars that multiply the elongation rate to obtain initiation and termination rates, respectively. For ease of simulation, the enzyme enters the simulation one nucleotide at a time, and exits the simulation by hopping off as a unit.

### ETAM

The torque interaction between RNAPs described in the Results section above, allows the polymerases to work together using the over-twisting and under-twisting in the DNA strand. The following section makes this process more precise by deriving a mathematical expression for the amount of torque that an RNAP imparts to DNA during translocation.

#### Torque between RNAP and DNA

DNA consists of two strands in a double helix structure. In addition, DNA is a flexible polymer structure that can experience bend. A polymer’s bend-persistence length is a mechanical property that characterizes its stiffness. For lengths less than the bend-persistence length, a polymer such as DNA, will exhibit behavior similar to that of an elastic rod. The bend-persistence length of DNA is estimated to be 150 bp [37]. Therefore, on length scales shorter than the persistence length, the DNA strand is comparable to an elastic rod. Since the average distance between elongating polymerases on an rrn gene is 100 bp [38], it is reasonable to assume that, on average, the force exerted by one elongating RNAP on its neighbors occurs over a distance of approximately 100 bp. Since this distance is within the persistence length reported in the literature, we assume that the local behavior of the DNA strand connecting two adjacent RNAPs can be modeled as an elastic rod. The movement of the RNAP enzyme in the ETAM model is the same as in the TASEP model, where the entire enzyme moves forward one nucleotide at a time as a unit. In addition, the footprint of an RNAP is approximately 35 bp, of which 17 bp is occupied by the transcription bubble [39]. As depicted in Fig 1, we assume that the 17 bp where DNA is unwound inside of the RNAP are anchored and cannot be twisted but that the other 18 bp are free to twist under an appropriate force. We assume that these 18 base pairs are partitioned into 9 bp on either side of the bubble. That is, the 9 bp of the strand that are outside of the transcription bubble of *P*_*i*_ yet still covered by the front end of the RNAP are considered to be part of the elastic rod located between *P*_*i*_ and its leading RNAP and are susceptible to twisting forces. Likewise, the 9 bp covered by the rear end of the RNAP are considered to be part of a separate elastic rod located between *P*_*i*_ and its trailing RNAP and are susceptible to twist. In this setting, we use classical elasticity theory to describe the interaction between a small segment of the DNA strand (elastic rod) and an elongating polymerase. The mass of the RNAP itself is not accounted for in the current version of the model, and the torque calculation is only based on the elongation motion of the RNAP and the amount of torsion imparted on the rod by that motion.

**Fig 6:**
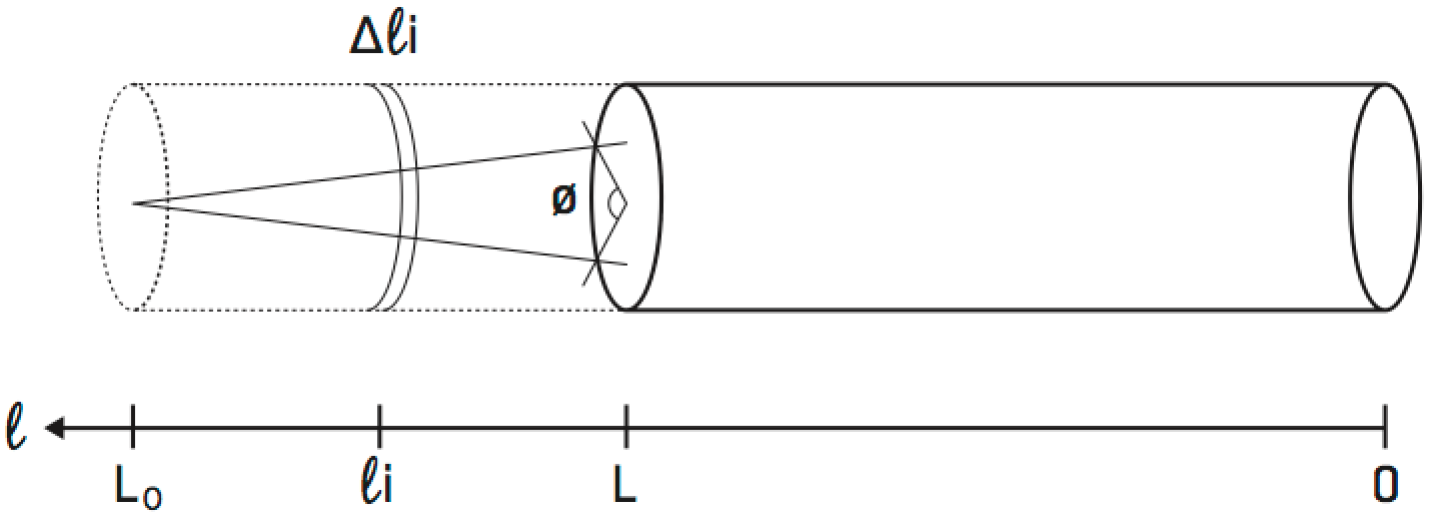
An elastic rod under torsion. The portion of the cylinder that is a dashed line represents the original distance *L*_0_ and the current distance *L*. As the distances decreases from *L*_0_ to *L*, the total amount of twist added to the rod is *ϕ*. The small increment Δ*ℓ*_*i*_ represents a change in length due to RNAP motion by one nucleotide.

The torque $\hat{\tau }$ stored in an elastic rod under torsion is

$$\hat{\tau }=\mu \frac{\pi r^{4}}{2L}\Delta \phi$$ (1)

where *r* is the radius (≈ 1 nanometer (nm) for DNA [37]), *μ* is the shear modulus, *L* is the length of the rod, and Δ*ϕ* is the angle of the total twist [40], see Fig 6. All quantities representing length are measured in base pairs and converted into nanometers using the conversion 1 bp = 0.34 nm [37].

In the context of our model, there is some flexibility in how one associates the length of the rod *L* with the distance between two neighboring polymerases as shown in Fig 1. First note that Eq (1) indicates that the torque goes to infinity as *L* → 0. In terms of our model, this would preclude direct contact between two polymerases. However, it has been observed experimentally [41] that RNAPs periodically exhibit direct contact with their neighbors during transcription. In addition, we recall that the footprint of elongating RNAP is approximately 35 bp, of which 17 bp is occupied by the transcription bubble [39]. The 17 bp inside of the transcription bubble cannot be twisted by an elongation force, but the remaining 18 bps are free to twist under an appropriate force. Therefore even when two RNAPs are directly adjacent to each other (without any empty base pairs separating them), there are still 18 bp between their corresponding transcription bubbles. Hence, we define the length *L* in Eq (1) to be the distance between the transcription bubbles of adjacent RNAPs, where *L* has units of nucleotides. This ensures that the underlying mathematical quantity in Eq (1) remains bounded.

The shear modulus, *μ*, is calculated using the formula

$$\mu =\frac{Y}{2(1+\nu )}$$ (2)

where *Y* is Young’s modulus and *ν* is Poisson’s ratio [40]. The Poisson ratio of DNA has been reported to be anywhere in the interval *ν* ∈ [−0.7, 0], for more details see [42–44]. To calculate this parameter specifically we will follow the paper by Manning et al [44]. Using the formula

ν=B/C-1 (3)

where *B* is the bending modulus and *C* is the twisting modulus [44], we calculate *ν* ≈ −0.5. Using these values for the Poisson ratio and Young’s modulus in Eq (2), we have estimated the shear modulus for DNA to be

$$\mu \approx 300\;{\text{pN/nm}}^{2}.$$

We also simulated results using *ν* = 0 and *ν* = −0.25 and observed qualitatively similar behavior. Parameters values used in the simulations presented here along with corresponding literature references can be found in Table 3.

**Table 3:** Parameters used in calculation of torque.

Parameter	Symbol	Value	Reference
Shear Modulus	*μ*	300 pN/nm^2^	calculated using Eq (2)
Young’s Modulus	*Y*	300 MPa	[37]
Poisson Ratio	*ν*	-0.5	calculated using Eq (3)
Bending Modulus	*B*	230 pN nm^2^	[44]
Twisting Modulus	*C*	460 pN nm^2^	[44]

Next we derive a mathematical equation which describes the relationship between the length of the flexible (rod) segment of DNA strand between the transcription bubbles of successive RNAPs and the amount of torque in that segment. This segment of the strand is modeled using an elastic rod as sketched in Fig 6, and the notation is made precise below. Because there are 10.5 base pairs in one full helical rotation of DNA, the twist angle Δ*ϕ* in Eq (1) is proportional to the change in length Δ*ℓ* as

$$\Delta \phi =\frac{2\pi }{10.5}\Delta \ell .$$ (4)

The torque between *P*_*i* − 1_ and *P*_*i*_ (see Fig 1) due to the accumulation of applied torque is calculated by adding small increments of torque that correspond to motion by one nucleotide as in Eq (4). This measure of torque is always done in relation to the state of neutral twist of that segment of the strand. To be precise, we consider the segment of DNA strand between *P*_*i* − 1_ and *P*_*i*_ as an elastic rod (see Fig 1), and we assume that the DNA strand is in a state of neutral twist (not over-twisted or under-twisted) at the time of initiation and so the torque is zero. When the trailing RNAP (*P*_*i*_) initiates, the distance between *P*_*i*_ and the nearest leading RNAP (*P*_*i* − 1_) downstream (in the direction of elongation) is defined to be *L*_0_. Assume that at some later time, the distance between *P*_*i* − 1_ and *P*_*i*_ is *L*. In order to derive the total amount of torque stored in the rod at this instance, one notes that the amount of torque between *P*_*i* − 1_ and *P*_*i*_ can be computed by treating the segment of the strand as an elastic rod with an original length of *L*_0_ that has experienced a twist corresponding to angle *ϕ*, with a resulting length of the rod denoted by *L*, see Fig 6. The total torque is calculated by adding small increments of torque that correspond to elongation by one nucleotide.

$$\Delta \tau_{i}=\mu \frac{\pi r^{4}}{2\ell_{i}}\Delta \phi_{i}=\mu \frac{\pi r^{4}}{2\ell_{i}}\left(\frac{2\pi }{10.5}\Delta \ell_{i}\right).$$ (5)

In order to construct a model that is efficient for extensive and repeated simulation, we use a continuous approximation of Δ*τ*_*i*_ by *dτ*, and lengths *ℓ*_*i*_ by *s*. The total torque in a segment of DNA strand with initial length *L*_0_ and final length *L* is approximated by

$$\tau =\tau \left(L\right)=\int_{L}^{L_{0}}d\tau =\int_{L}^{L_{0}}\mu \frac{\pi r^{4}\left(\frac{2\pi }{10.5}\right)}{2s}\;ds=\frac{\mu \pi^{2}r^{4}}{10.5}\ln\left(L_{0}/L\right).$$ (6)

Note that for *L* < *L*_0_ the DNA is over-twisted and the torque is positive, as in Fig 6. Conversely, if *L* > *L*_0_ the DNA is under-twisted and the torque is negative. These correspond to the resisting and assisting torque found in [24]. If *L* = *L*_0_ the torque is zero, since the DNA has the same length as when the trailing polymerase initiated onto the DNA. With this formula for the torque between two neighboring RNAPs, we next describe how this formula is used within the context of the stochastic elongation model.

#### Incorporating torque into the stochastic model

We will number RNAPs by index *i* which denotes the order of their initiation. The *i*-th polymerase will be characterized in the model by three numbers

$$P_{i}=\left(n_{i},L_{0,i},T_{i}\right)$$ (7)

where *i* represents a positive integer. This triple will be updated each time the RNAP translocates along the strand. The term *n*_*i*_ is an integer value denoting the furthest downstream nucleotide number occupied by the *i*th RNAP, while *T*_*i*_ is the time of the next elongation of *P*_*i*_. The calculation of this value is addressed in the section entitled Incorporation of Pauses. Upon initiation of *P*_*i*_ onto the strand, define *L*_0,*i*_ to be the distance (measured in nucleotides) between the transcription bubbles of *P*_*i*_ and that of its leading RNAP, *P*_*i* − 1_, at the time that *P*_*i*_ initiates transcription. This distance is calculated and stored in the triplet for each RNAP. When *P*_*i*_ initiates, the value of *L*_0,*i*_ is fixed, however, this distance may be different for each RNAP as its initiation occurs at a randomly generated time and is independent of the distance traveled by any previously initiated RNAPs. We denote *L*_*i*_(*t*) to be the distance between the transcription bubbles of *P*_*i*_ and its leading RNAP *P*_*i* − 1_ at time *t*. This distance is computed by accessing the variables *n*_*i* − 1_ and *n*_*i*_ of *P*_*i* − 1_ and *P*_*i*_ and measuring their difference. Using this distance, we can calculate the length of the DNA strand that is free to twist by subtracting the length of the transcription bubble, 17 nts. In other words,

$$L_{i}=\left(n_{i-1}-n_{i}\right)-17.$$ (8)

In order to quantify the role of the torque calculation, we begin by considering elongation. First, suppose there are three RNAPs positioned on a segment of the DNA strand, and denote these RNAPs as *P*_*k*_, for *k* = *i* − 1, *i*, *i*+1 as labelled in Fig 1, and further suppose that *P*_*i*_ has just experienced elongation. The values *L*_*i*_ and *L*_*i*+1_ are calculated immediately following elongation. Using these distances, as well as *L*_0,*i*_, and *L*_0,*i*+1_, the torque that *P*_*i*_ is currently experiencing is calculated using Eq (6) in the following manner. Specifically, the torque has two components. The first is the component that corresponds to the torque between *P*_*i*_ and *P*_*i* − 1_, denoted *τ*(*L*_*i*_), and the torque between *P*_*i*_ and *P*_*i*+1_, denoted by *τ*(*L*_*i*+1_). Define *τ*_*i*_ to be the total torque experienced by polymerase *P*_*i*_. It is calculated as follows

$$\tau_{i}=\tau \left(L_{i}\right)-\tau \left(L_{i+1}\right)=\frac{\mu \pi^{2}r^{4}}{10.5} [\ln\left(\frac{L_{0,i}}{L_{i}}\right)-\ln\left(\frac{L_{0,i+1}}{L_{i+1}}\right)].$$ (9)

Analogously, calculations are repeated for RNAPs *P*_*i* − 1_ and *P*_*i*+1_, recalculating the torque for all three RNAPs after translocation of *P*_*i*_. It is important to note, the calculations outlined in the previous paragraphs are only performed for the affected RNAPs; the elongating RNAP and its neighboring RNAPs.

During transcription, RNAPs first experience initiation, and the measurement of torque for initiation can be interpreted as a special case of the previous discussion. Upon initiation of an RNAP, labeled *P*_*i*_ in Fig 1, the distance between *P*_*i*_ and its leading RNAP, the quantity *L*_0,*i*_, is set. Upon initiation and subsequent elongation of *P*_*i*_, the torque behind this polymerase is zero until the next initiation. Once a trailing RNAP, *P*_*i*+1_ here, has initiated onto the strand, the calculation of the torque *τ*_*i*_ will have nonzero contributions from both neighbors of the polymerase.

We model RNAP termination as follows. Again referencing Fig 1, suppose *P*_*i* − 1_ is the RNAP that is closest to the termination end of the DNA strand and therefore will be the next polymerase to terminate. Prior to termination, the torque measure *τ*_*i* − 1_ has a nonzero contribution only from *τ*(*L*_*i*_). That is *τ*_*i* − 1_ = 0 − *τ*(*L*_*i*_). Likewise, during this situation *τ*_*i*_ has contributions from both neighboring RNAPs as long as *P*_*i* − 1_ is still transcribing the strand. Upon termination of *P*_*i* − 1_, the torque downstream of *P*_*i*_ is zero, hence *τ*_*i*_ = 0 − *τ*(*L*_*i*+1_). Since *P*_*i*_ is now the last RNAP on the DNA strand, *τ*_*i*_ will be calculated as such until *P*_*i*_ itself terminates.

#### Physical interpretation of torque

The formula for *τ*_*i*_ in Eq (9) is based on the total amount of both assisting and resisting torque that is experienced by polymerase *P*_*i*_. Here we give some physical insights into the positioning of the RNAP relative to each of its neighbors and its effect on the torque calculation. Recall that when the distance *L*_*i*_ is compared to the original distance *L*_0,*i*_, the DNA between them can be either under-twisted, over-twisted or neutral. Over-twisting in front of an RNAP provides a resisting torque, while over-twisting behind an RNAP provides an assisting torque (“push from the back”). On the other hand, under-twisting in front of an RNAP provides an assisting torque (“pull from the front”), while under-twisting in back of an RNAP is a resisting torque. Examining Eq (9) for several possible scenarios gives us insight into the influence of torque on movement of the polymerase.

**Fig 7:**
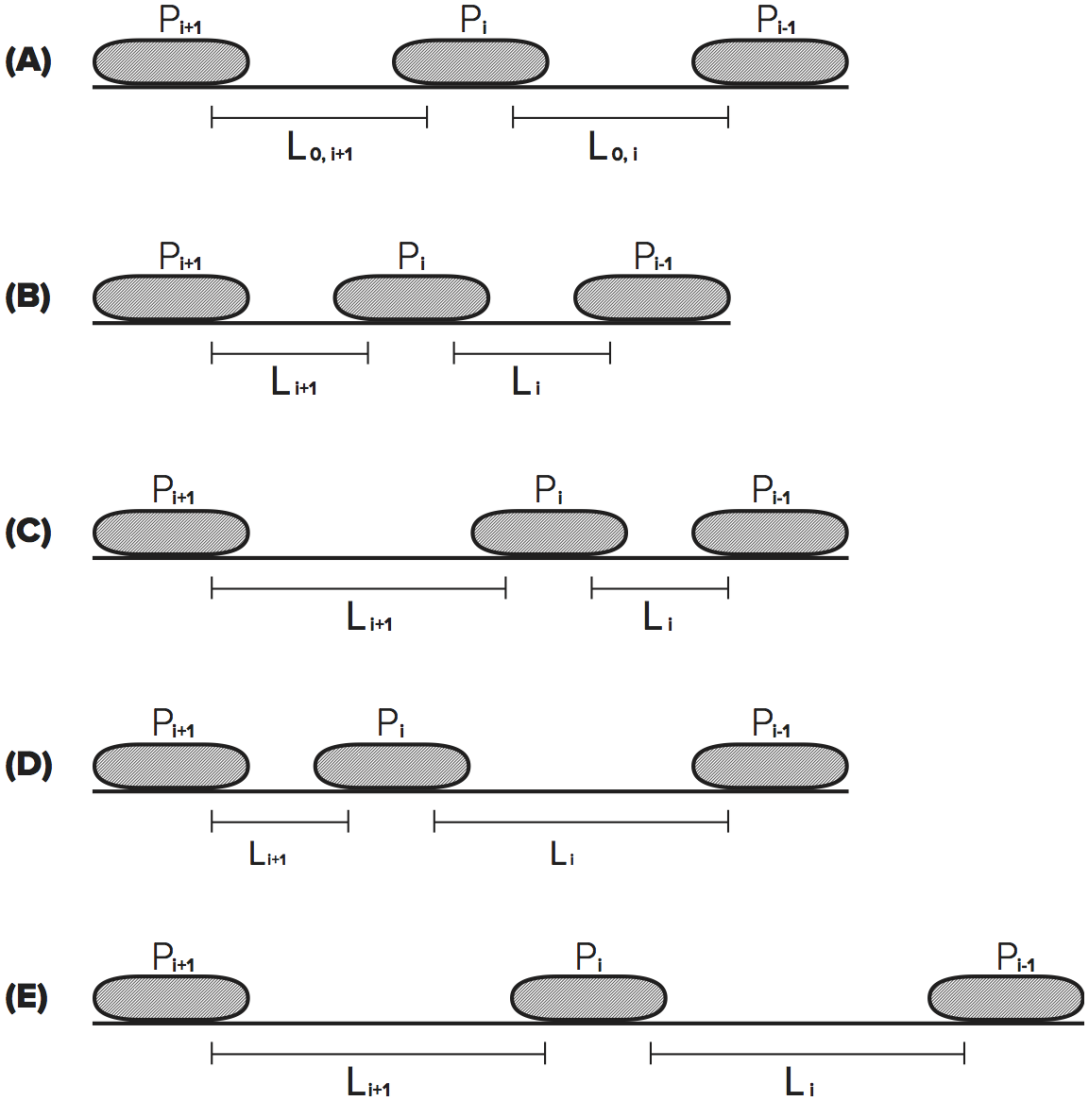
This figure depicts the four different cases of RNAP *P*_*i* − 1_, *P*_*i*_, and *P*_*i*+1_. Fig 7A shows the three RNAPs with the original distances between them. Then there are four different configurations; Fig 7B, *L*_0,*i*_ > *L*_*i*_ and *L*_0,*i*+1_ > *L*_*i*+1_, Fig 7C, *L*_0,*i*_ > *L*_*i*_ and *L*_0,*i*+1_ < *L*_*i*+1_, Fig 7D, *L*_0,*i*_ < *L*_*i*_ and *L*_0,*i*+1_ > *L*_*i*+1_, and Fig 7E, *L*_0,*i*_ < *L*_*i*_ and *L*_0,*i*+1_ < *L*_*i*+1_.

For the neutral case when *L*_0,*i*_ = *L*_*i*_ in Eq (9), *τ*(*L*_*i*_) is zero and there is no contribution to *τ*_*i*_ from that component. The contribution to *τ*_*i*_ is similar for the case of *L*_0,*i*+1_ = *L*_*i*+1_. Next we focus the discussion on the cases when *τ*(*L*_*i*_) and *τ*(*L*_*i*+1_) are nonzero. There are four cases to consider which are depicted in Fig 7, scenarios (B)—(E) and are discussed below.

1. *L*_0,*i*_ > *L*_*i*_ and *L*_0,*i*+1_ > *L*_*i*+1_
  The values of the two components of torque *τ*_*i*_ are *τ*(*L*_*i*_)>0 and *τ*(*L*_*i*+1_)>0. A positive value of *τ*(*L*_*i*_) corresponds to a resisting torque being experienced by polymerase *P*_*i*_ relative to the position of its leading RNAP. A positive value of *τ*(*L*_*i*+1_) provides an assisting torque for *P*_*i*_. Therefore the subtraction of *τ*(*L*_*i*+1_) implies that *τ*_*i*_ < *τ*(*L*_*i*_). In other words, RNAP *P*_*i*+1_ is assisting *P*_*i*_ to overcome the resisting torque in front of it at its current position.
2. *L*_0,*i*_ > *L*_*i*_ and *L*_0,*i*+1_ < *L*_*i*+1_
  The values of the two components of torque *τ*_*i*_ are *τ*(*L*_*i*_)>0 and *τ*(*L*_*i*+1_)<0. These two factors indicate that the DNA between *P*_*i*_ and *P*_*i* − 1_ is over-twisted and the DNA between *P*_*i*+1_ and *P*_*i*_ is under-twisted resulting in *P*_*i*_ experiencing resisting torques from both sides. The negative value of *τ*(*L*_*i*+1_) is subtracted, increasing the value of *τ*_*i*_, therefore the subtraction of *τ*(*L*_*i*+1_) implies that *τ*_*i*_ > *τ*(*L*_*i*_).
3. *L*_0,*i*_ < *L*_*i*_ and *L*_0,*i*+1_ > *L*_*i*+1_
  The values of the two components of torque *τ*_*i*_ are *τ*(*L*_*i*_)<0 and *τ*(*L*_*i*+1_)>0. The DNA between *P*_*i*_ and *P*_*i* − 1_ is under-twisted and the DNA between *P*_*i*+1_ and *P*_*i*_ is over-twisted resulting in *P*_*i*_ experiencing and assisting torque from both sides.
4. *L*_0,*i*_ < *L*_*i*_ and *L*_0,*i*+1_ < *L*_*i*+1_
  The values of the two components of torque *τ*_*i*_ are *τ*(*L*_*i*_)<0 and *τ*(*L*_*i*+1_)<0. The DNA between *P*_*i*_ and its neighboring RNAPs is under-twisted. This leads to *P*_*i*_ experiencing an assisting torque from the front and a resisting torque from behind.

### Mean Field Approximation Model

The role of a mean field approximation model, as for any other model, is to provide insight into a more detailed stochastic model. In deriving such a model, we inevitably simplify the fine model and therefore the deterministic model will preserve only some aspects of the detailed model. Some portions of the analysis given here use techniques borrowed from the treatment of various traffic flow models, and we point out a connection between this discussion and a particular traffic flow model in a remark at the end of this section.

Our starting point in deriving the mean field approximation model is the expression for torque given by Eq (9). Our goal is to capture behavior of small perturbations around the stochastic equilibrium when this torque is zero. This implies that there exists a *preferred distance* *L*_0_ such that *L*_0,*i*_ = *L*_0,*i*+1_ = *L*_0_ for all *i* and where *L*_*i*_ = *L*_*i*+1_ = *L*_0_. Assume that the elongation velocity at zero torque is *β*. It follows that the polymerase *P*_*i*_ is elongating with a velocity

$$\frac{dP_{i}\left(t\right)}{dt}=V\left(\tau_{i}\left(t\right)\right)=V\left(\frac{\mu \pi^{2}r^{4}}{10.5}\ln\left(\frac{L_{i+1}}{L_{i}}\right)\right),$$ (10)

where the torque *τ*_*i*_ at time *t* is given by Eq (9), and the expression inside the logarithm is simplified by our assumption that *L*_0,*i*_ = *L*_0,*i*+1_ = *L*_0_ for all *i*. The velocity function *V* is computed using one of the polynomial functions given by either Eqs (19) or (22) introduced in the section Incorporating Experimental Data to Determine the Effect of Torque. In particular, simulation results presented in the section Linear Fit to the Data show that a (piecewise) linear relationship between torque and velocity is sufficient to observe the cooperative behavior among RNAPs that is the subject of this work. Using a linear relationship between torque and velocity, say $V\left(\tau \right)=\tilde{A}\tau +\tilde{B},$ where $\tilde{A},\tilde{B}$ represent arbitrary constants, the form of the velocity described above becomes.

$$\frac{dP_{i}\left(t\right)}{dt}=A\ln\left(\frac{L_{i+1}}{L_{i}}\right)+B,$$ (11)

where *A*, *B* are constants that are determined by the equation in Eq (9) as well as the piecewise linear velocity relationship used here which is found in Eq (22). Note that the constant *A* < 0 since the relationship between torque and velocity is a strictly decreasing one. In order to work with all positive constants, we let *C* = −*A* and using basic rules of logarithms, we arrive at the relationship

$$\frac{dP_{i}\left(t\right)}{dt}=C\ln\left(\frac{L_{i}}{L_{i+1}}\right)+B,$$ (12)

where *C*, *B* > 0.

Next we examine the quotient *L*_*i*_/*L*_*i*+1_ in view of our assumption that both distances *L*_*i*_ and *L*_*i*+1_ are near the preferred spacing *L*_0_. Note that if both RNAPs are exactly at the preferred spacing, then *L*_*i*_ = *L*_*i*+1_ = *L*_0_, and the velocity simplifies to the constant *B*. Hence we prescribe *B* = *β*. Now suppose that the RNAPs are near the preferred spacing but not precisely achieving that spacing. Noting that these distances represent consecutive distances surrounding the RNAP on the DNA strand, if the RNAP elongates one nucleotide, then one distance *L*_*i*_ is perturbed just slightly from the steady state, and the other is perturbed by that same amount but with the opposite sign. That is, the quotient can be expressed as

$$\frac{L_{i}}{L_{i+1}}=\frac{L_{0}+s}{L_{0}-s}=\frac{\left(L_{0}+s\right)}{L_{0}} [\frac{1}{1-\frac{s}{L_{0}}}]=\left(1+\frac{s}{L_{0}}\right) [\frac{1}{1-\frac{s}{L_{0}}}]$$

where |*s*|< < 1 is a small parameter. Using a series expansion for the term in the square brackets, we have

$$\frac{L_{i}}{L_{i+1}}=\left(1+\frac{s}{L_{0}}\right) [1+\left(\frac{s}{L_{0}}\right)+{\left(\frac{s}{L_{0}}\right)}^{2}+\cdots ]=1+2\left(\frac{s}{L_{0}}\right)+O{\left(\frac{s}{L_{0}}\right)}^{2}$$

Returning to the ODE system above, we make the linear approximation of the quotient in terms of *s* so that the RNAP velocity is approximately

$$\frac{dP_{i}\left(t\right)}{dt}=C\ln\left(\frac{L_{i}}{L_{i+1}}\right)+\beta =C\ln\left(1+2\frac{s}{L_{0}}\right)+\beta$$ (13)

Next we proceed to relate the expression $\frac{s}{L_{0}}$ to the steady state density of the system so that the left side of Eq (13) is replaced by the steady state RNAP velocity. We use a typical convention from the traffic flow literature and assume that the density is inversely proportional to distance between polymerases $\rho =\frac{1}{L}$. Assuming that the RNAP density, *ρ* is either at steady state or is a slight perturbation from steady state, we write

$$\rho =\frac{1}{L}=\frac{1}{L_{0}+s}=\frac{1}{L_{0}}\left(\frac{1}{1+\frac{s}{L_{0}}}\right),$$

Using a series expansion for the term in parentheses, one obtains

$$\rho =\frac{1}{L_{0}}\left(1-\frac{s}{L_{0}}+O{\left(\frac{s}{L_{0}}\right)}^{2}\right).$$

Approximating the density with the linear term, we have

$$\frac{1}{L}\approx \frac{1}{L_{0}}\left(1-\frac{s}{L_{0}}\right)=\frac{1}{L_{0}}-\frac{s}{L_{0}^{2}}$$

which then simplifies to

$$\frac{s}{L_{0}}\approx L_{0}\left(\frac{1}{L_{0}}-\frac{1}{L}\right)=L_{0}\left(\rho_{0}-\rho \right).$$

Using this relation in Eq (13), when $\rho =\rho_{0}=\frac{1}{L_{0}}$, then we have that the average velocity

$$v\left(\rho \right)=C\ln\left(1+2L_{0}\left(\rho_{0}-\rho \right)\right)+\beta .$$ (14)

**Fig 8:**
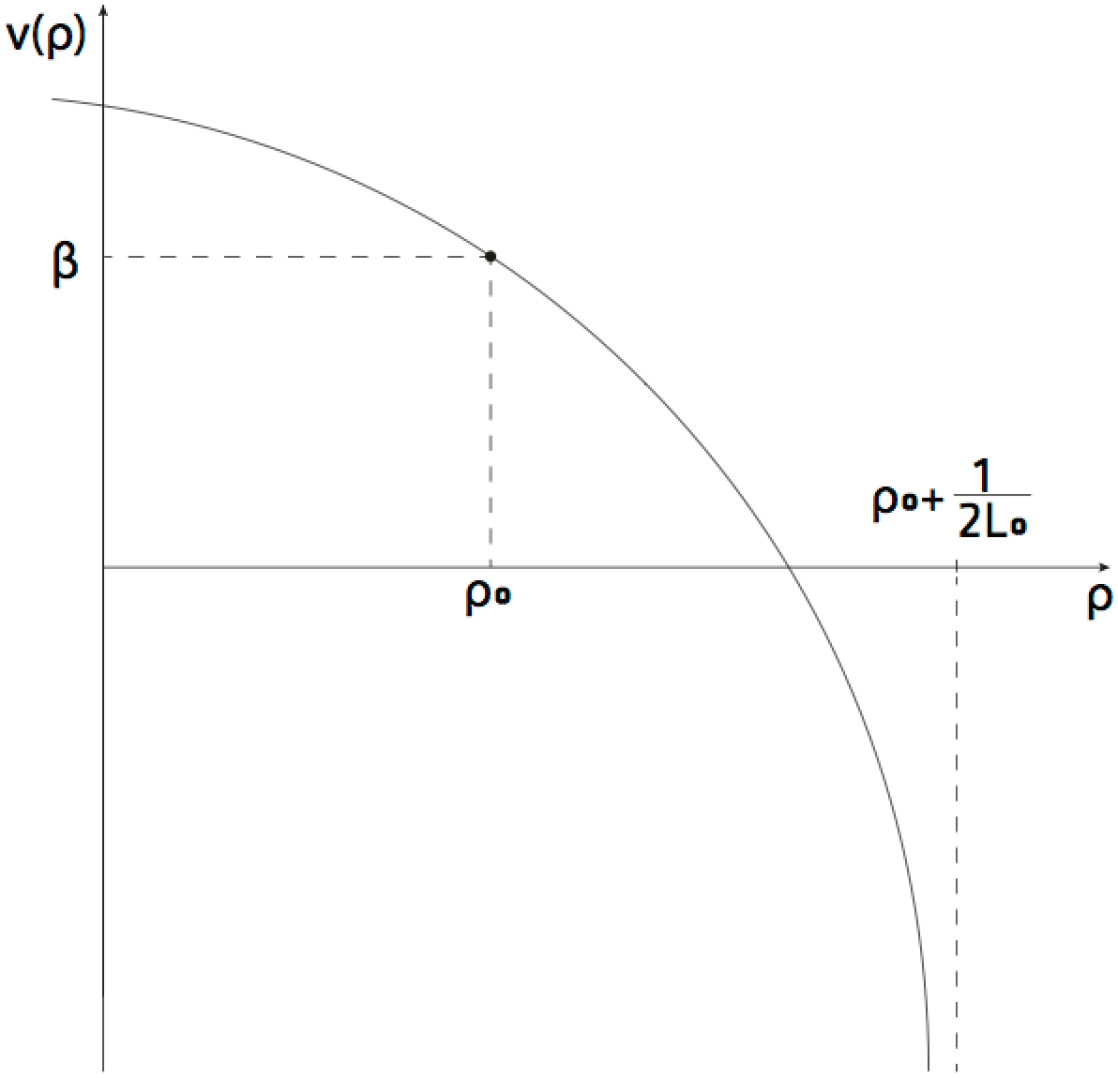
Sketch of the general functional behavior of the mean field velocity prediction of the torque model.

Observe, that when *ρ* = *ρ*_0_ the RNAP velocity is *β*. However, for small perturbations of *ρ* so that *ρ* > *ρ*_0_ then the argument in the logarithm is less than 1 and the particle velocity is lower than *β*, and if *ρ* < *ρ*_0_ then the argument in the logarithm is greater than 1 and the particle velocity is faster that *β*. This self-adjustment of the velocity is a negative feedback that rejects local perturbations away from preferred density *ρ*_0_ and preferred velocity *β*. That is,

$$v^{\prime }\left(\rho \right)=\frac{-2CL_{0}}{1+2L_{0}\left(\rho_{0}-\rho \right)},$$

and *v*′(*ρ*_0_)<0, which can be observed graphically in Fig 8.

#### Remark

The model given by Eq (12) has an interesting connection to a classical car-following model [45–48]. When the classical car-following model is derived by accounting for car velocities of both the leading and trailing neighbors, then this choice captures the mathematical form of velocity that we derived using the torque formulation from the elastic rod equations and the linear torque to velocity relationship within the ETAM model. In addition, the car-following model provides us with an attractive analogy between a string of polymerases transcribing a DNA strand which synchronize their motion through torque, and a string of driverless electronically synchronized cars. The power of this analogy is limited by the fact that the car-following model that we begin with is second order and thus the inertial term is important, which is not the case for the mean field model that we ultimately derive for ETAM. The following paragraphs give a short overview of the modifications made to the classical car-following model in order to observe the type of cooperative interactions between both leading and trailing polymerases discussed above.

Let *P*_*i*_(*t*) denote the position of the *i*-th vehicle on a straight, single lane roadway. For the classical car-following model, the acceleration of the car can be described by the equation

$$\frac{d^{2}P_{i}\left(t\right)}{dt^{2}}=-\left(\frac{C}{P_{i-1}-P_{i}}\right)\left(\frac{dP_{i}\left(t\right)}{dt}-\frac{dP_{i-1}\left(t\right)}{dt}\right),$$ (15)

where the acceleration depends inversely on the distance between itself and the car directly ahead of it, (the *i* − 1st car on the roadway), *L*_*i*_: = *P*_*i* − 1_ − *P*_*i*_, as defined in the section Incorporating Torque in the Stochastic Model, and the velocity of *P*_*i*_ relative to its leading car. The constant *C* > 0 is related to the specific ability of the driver to react to the changing conditions in the traffic.

In order to complete the connection with the mean field approximation for the ETAM model, we adjust the car following model from Eq (15) in two fundamental ways. First, in ETAM the polymerase velocity is affected by the leading polymerase as well as the trailing polymerase. Second, the car following model does not have apriori a preferred distance *L*_0_ at which the forces from two flanking cars are zero, and as a result there is no acceleration of the car in the middle. To address the first issue, we expand the model by including a force due to the trailing car effect into Eq (15) so that the new equation is written

$$\frac{d^{2}P_{i}\left(t\right)}{dt^{2}}=-\left(\frac{C}{P_{i-1}-P_{i}}\right)\left(\frac{dP_{i}\left(t\right)}{dt}-\frac{dP_{i-1}\left(t\right)}{dt}\right)+\left(\frac{C}{P_{i}-P_{i+1}}\right)\left(\frac{dP_{i+1}\left(t\right)}{dt}-\frac{dP_{i}\left(t\right)}{dt}\right).$$ (16)

We integrate using substitutions with

$$u=P_{j-1}\left(t\right)-P_{j}\left(t\right)\;\text{and}\;du= [\frac{dP_{j-1}\left(t\right)}{dt}-\frac{dP_{j}\left(t\right)}{dt}]dt,$$

for *j* = *i*, *i*+1 to obtain the expression for velocity

$$\begin{array}{ccc} \frac{dP_{i}\left(t\right)}{dt} & = & C\ln\left(P_{i-1}-P_{i}\right)-C\ln\left(P_{i}-P_{i+1}\right)+d_{i}\\ & = & C\ln\left(\frac{P_{i-1}-P_{i}}{P_{i}-P_{i+1}}\right)+d_{i}\\ & = & C\ln\left(\frac{L_{i}}{L_{i+1}}\right)+d_{i}. \end{array}$$

One immediately observes that if the distances between consecutive RNAPs is approximately the average *L*_0_, then the logarithmic term in the above equation vanishes. Moreover, if the constant is set to the average velocity *d*_*i*_ = *β*, then the expression given above for the car velocity has exactly the same algebraic form as that of the mean field approximation derived for the ETAM model in Eq (12).

Now that a theoretical justification of the cooperative behavior seen in the ETAM model has been discussed, we discuss the effects of torque on transcriptional pauses, specifically the ubiquitous pauses described previously. The discussion below gives the last concepts needed for incorporating torque into the stochastic model.

### Incorporating Experimental Data to Determine the Effect of Torque

**Fig 9:**
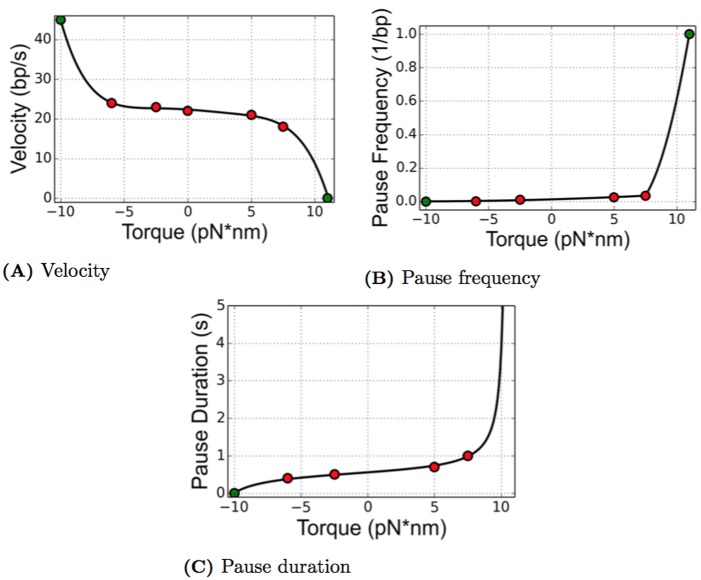
The data published by Ma et. al. is presented (red dots) as well as the curve fit to the data (black). The values that we choose based on biological information for very high and very low torque values are shown also (green dots). (A) shows the velocity as a function of torque, while pause frequency and pause duration as a function of torque are given by (B) and (C) respectively. The equation for each curve can be found in Eqs (19)–(21), respectively.

Recent single molecule experiments by Ma and co-authors [24] attempt to advance our understanding of the relationship between the torque and the movement of the RNAP. The data obtained in those experiments suggests a relationship between torque and the elongation velocity of the RNAP. It has also been known for many years that during elongation, RNAPs experience short, frequent pauses where active elongation is stalled or arrested. Ma [24] also suggests that the nature of these pauses is tied to the amount of torque that an RNAP is experiencing at a given moment of time. The data reported in [24] was limited, as can be seen in Fig 9A–9C, and we explored various ways to develop accurate representations of these relationships that could be used in a mathematical model. The following subsections give an overview of various approaches to incorporating the data into the current model, and simulation results using these different choices are discussed later in the paper.

#### Incorporating experimental data into the ETAM model

For the ETAM model, we first fit the data from [24] to extract a functional relationship between the torque experienced by an individual polymerase and its elongation velocity, pause frequency and pause duration. Results from various biological experiments describing physical properties of DNA are used. For instance, the full model employs a stall torque, a high torque value experienced by RNAPs where they are unable to elongate, reported in [24] to be on average 11 pN⋅nm. At values close to melting torque -10 pN⋅nm, the RNAPs would be experiencing a large amount of assistance, therefore we assumed a high velocity with no pauses. While there are many classes of functions to fit data, our goal was to fit the data using low order polynomials. We use the notation *V*(*τ*) to denote the polynomial function used to describe the elongation velocity, *F*(*τ*) to denote the frequency of occurrence of a pause as a function of torque, and *D*(*τ*) to denote the duration time of a given pause.

One of the first papers to experimentally measure the force-velocity relationship was Wang et. al. [25]. They measured velocity as a function of progressively larger positive forces that were applied through a feedback-controlled optical trap, and the authors obtained the theoretically justified expression

$$v\left(F\right)=\frac{v_{0}\left(1+A\right)}{1+A\text{exp}(F\delta /k_{B}T)}$$ (17)

where *A* is a dimensionless constant that determines the degree to which either mechanical or biochemical events limit the enzymatic cycle at vanishing load and *δ* is a characteristic distance. After non-dimensionalizing Eq (17) [25], the following relationship obtained for normalized velocity $V:=\frac{v}{v_{0}}$

$$V\left(f\right)=\frac{1+A}{1+A\text{exp}(-f\ln(A))}\approx \frac{1}{1+a^{f-1}}$$ (18)

where *f* > 0 is the dimensionless force, and *a* = *A*^−1^. Since in our model we consider both positive and negative torque, we assume that for negative *f* we have

V(-f)≈-V(f),f>0.

Finally, for simplicity of the fitting procedure we approximate *V*(*f*) by a relatively low degree fifth order polynomial, which still captures strong nonlinear behavior of velocity described by Eq (18).

When constructing the function *V*(*τ*) describing the elongation velocity, we assume that the velocity would decrease to 0 nt/s at the stall torque 11 pN⋅nm. To impose a value of velocity when torque was -10 pN⋅nm, we assume the polymerases would be traveling at rate that is significantly higher than *V*(0). This assumption is due to the fact that negative torque is assisting torque which would aid efficient elongation. A large amount of assisting torque would lead to extremely fast velocities. For simplicity, we choose *V*(−10) = 45 nt/s, which is approximately twice the velocity at a torque of 0 pN⋅nm. Using these two extra data points we had a good agreement between the velocity data and a fifth order polynomial.

$$V\left(\tau \right)=-0.0002\tau^{5}+0.0008\tau^{4}+0.0041\tau^{3}-0.035\tau^{2}-0.2166\tau +22.3574.$$ (19)

In order to develop equations for pause frequency and pause duration, we make assumptions that are consistent with the biology. At the stall torque we assume the RNAPs will pause with probability 1. Incorporating this information into the pause frequency function, an accurate fit is difficult to achieve using a single function since the data increases very quickly to 1 at a torque value of 11 pN⋅nm. Using an exponential fit severely underestimates the negative torque values, since the point (11, 1) dominates the fitting procedure. For this reason, a piecewise quadratic function is used to describe the functional relationship of the data.

$$F\left(\tau \right)=\left\{\begin{array}{cc} 0.0001\tau^{2}+0.0022\tau +0.0128 & \tau \leq 7.5\\ 0.0453\tau^{2}-0.5621\tau +1.7032 & \tau >7.5 \end{array}\right.$$ (20)

As the torque approaches the stall torque, the pause duration should increase rapidly. RNAPs are able to reenter active elongation after reaching a stall torque if the torque is alleviated but will stay in a pause if the torque remains at the stall torque [24]. To capture both the rapid rise and infinite duration at 11 pN⋅nm, we choose a function with a vertical asymptote at the stall torque. In order to produce the asymptotic behavior, we use a tangent function as given below.

D(τ)=0.1914tan((π/23)τ+0.1469)+0.5298 (21)

For a torque of -10 pN⋅nm, we impose a pause duration of 0 seconds. The original data from [24] and the curve fits can be seen in Fig 9.

Fig 9A–9C correspond to qualitatively realistic relationships between torque and the parameters influencing the motion of a polymerase. The case of a zero value of torque is considered to be the case of no resistance. When an RNAP experiences a resisting torque, this corresponds to a positive torque value. The resulting elongation velocity (see Fig 9A) decreases from that of the zero torque case so that elongation is hampered. In addition, as the amount of torque increases, the velocity of the motion decreases, and the forward motion of the RNAP is resisted. Examining Fig 9B–9C, both the pause frequency and pause durations are strictly increasing functions of torque. Pauses are more likely to occur when the polymerase experiences a resisting torque, and when a pause occurs, it is likely to be longer in duration if the torque is resisting forward motion. The figures also reflect the case that experiencing an assisting torque is associated with a large elongation velocity along with small pause frequency and pause duration for an individual RNAP.

#### Linear fit to the data

With limited data points, one may attempt a linear fit without using information such as stall torque. In addition to simulations of the data fits explained above, we also ran simulations using a piecewise linear curve fit. The equations are given in this section, and we discuss the results of these simulations in the section Results from the Piecewise Linear Data Fit. The data presented in [24] looks almost linear except for the highest torque value of 7.5 pN⋅nm. Therefore we choose to build a piecewise linear functional relationship with two linear fits, one using the data points at -6 pN⋅nm and 5 pN⋅nm, the other uses the data points at 5 pN⋅nm and 7.5 pN⋅nm. These equations are

$$V\left(\tau \right)=\left\{\begin{array}{cc} -0.2727\tau +22.3636 & \tau \leq 5\\ -1.2\tau +27 & \tau \geq 5 \end{array}\right.$$ (22)

$$F\left(\tau \right)=\left\{\begin{array}{cc} 0.002277\tau +0.013864 & \tau \leq 5\\ 0.004\tau +0.005 & \tau \geq 5 \end{array}\right.$$ (23)

$$D\left(\tau \right)=\left\{\begin{array}{cc} 0.027273\tau +0.563636 & \tau \leq 5\\ 0.12\tau +0.1 & \tau \geq 5 \end{array}\right.$$ (24)

**Fig 10:**
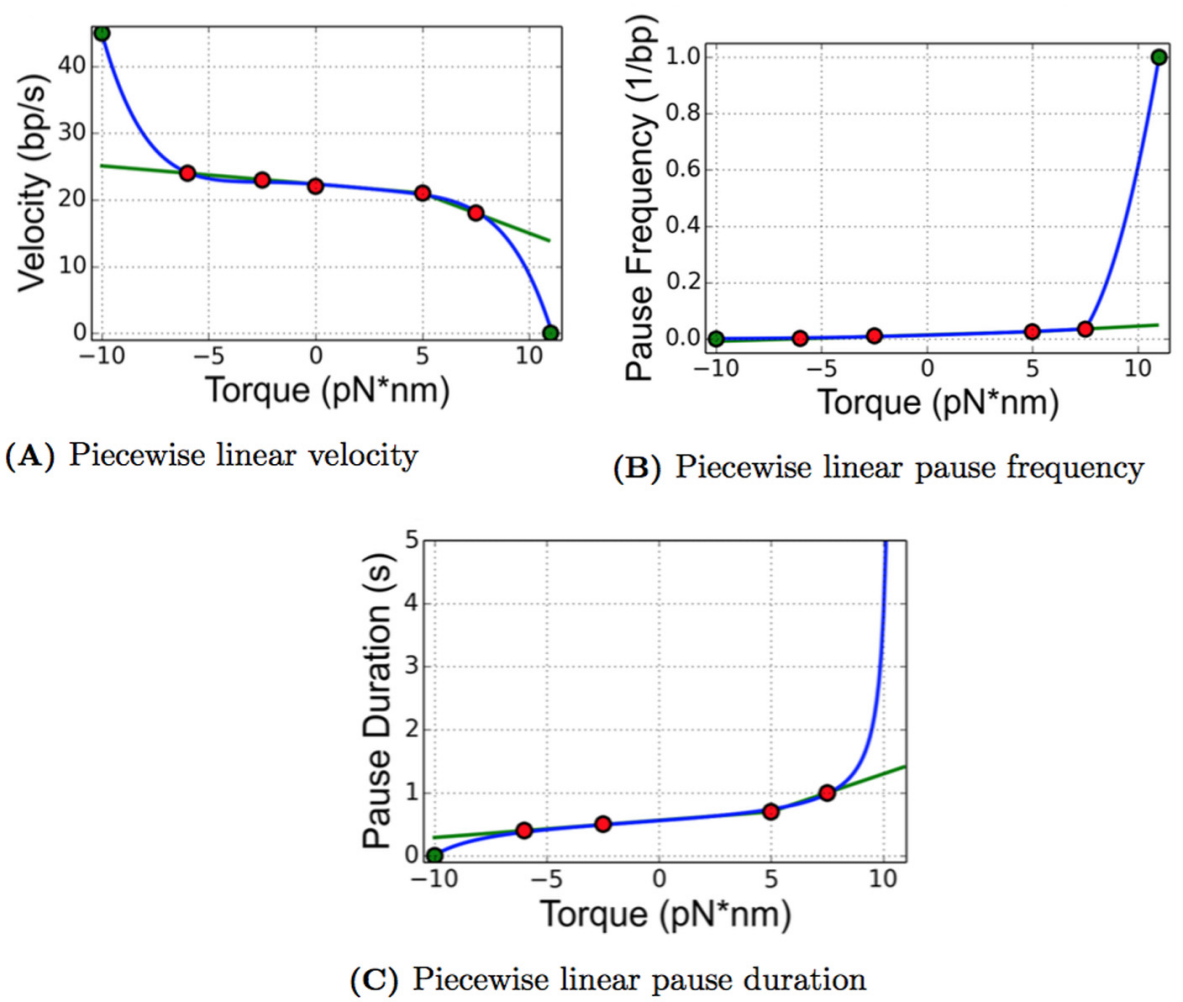
The data published by Ma et. al. is presented (red dots) as well as the piecewise linear curve fit to the data (green). The nonlinear polynomial fit is provided (blue) with our imposed values (green dots) for comparison purposes. As in Fig 9, the velocity is shown in (A), the pause frequency is presented in (B), and the pause duration is in (C). The equation for each piecewise linear curve can be found in Eqs (22)–(24), respectively.

The data points and piecewise linear curve fits, as well as the nonlinear curve fits for comparison purposes, can be found in Fig 10.

### Simulation

We simulate the transcription process using a Kinetic Monte Carlo algorithm [49, 50] which is often used for simulation of coupled Poisson processes. First, we provide an outline of the computations performed at each step of the simulation following an elongation event. The incorporation of the pauses and the recording of the collisions is detailed in this section, and we also describe the basic structure and order of events within the simulation algorithm.

#### Simulation events and implementation of pauses

The simulations consist of a series of events that occur for each RNAP within the system. These events are elongation and initiation. We assume that the time between two consecutive events for a given polymerase is exponentially distributed. Therefore, given that an event for polymerase *P*_*i*_ just occurred, the amount of time that will elapse prior to the next event for *P*_*i*_ has a probability distribution *P*(*t*) = *λe*^ − *λt*^, where *λ* > 0 is a rate parameter. The expected value of an exponential distribution is 1/*λ*, which means that, on average, the time between events is 1/*λ* seconds. The greater the value of *λ*, the more quickly the next event occurs, while events with smaller rate parameters will happen less frequently. Each of the two main RNAP events has its own unique rate parameter. The first parameter of interest is related to elongation, and it is denoted by *β*, where *β* is the average rate at which a single RNAP elongates from one base pair to the next on the DNA strand in the absence of any pauses or collisions with other RNAPs. This parameter has units of nucleotide per second. The basic elongation event has a rate parameter *β*, which implies that an elongation will happen every 1/*β* seconds on average.

The second rate parameter that governs the basic transcription process is the average initiation rate, *λ* = (α*β), where *α* ∈ (0, 1] is a constant value and *β* is the average elongation rate as described above. Here *α* is used to scale the elongation rate to a lower value causing initiation events to happen less frequently, on average every 1/(α*β) seconds. The value of *α* is fixed for each simulation. The incorporation of pauses into the model is achieved through modifications made dynamically to the elongation rate parameter. Next we describe the details of the simulation events, and for each pause event, we describe the adjustments that are made to the various rate parameters.

#### Incorporation of pauses

Continuing the notation used in previous sections, suppose that an elongation event has occurred for *P*_*i*_. Once the torques *τ*_*i*+1_, *τ*_*i*_, and *τ*_*i* − 1_ are recalculated for RNAPs in Fig 1, these values are used to update both the velocity and the pause frequency of all three RNAPs using Eqs (19) and (20) respectively. The velocity is used as the rate parameter, *λ* in the exponential distribution defined above. Specifically, once *τ*_*i*_, the torque experienced by *P*_*i*_, is calculated, the velocity and pause frequency, *V*(*τ*_*i*_) and *F*(*τ*_*i*_) are calculated using Eqs (19) and (20). The time of the next movement of *P*_*i*_ is now governed by the probability distribution *P*(*t*) = *V*(*τ*_*i*_)*e*^−*V*(*τ*_*i*_)*t*^ with an expected value of 1/*V*(*τ*_*i*_). The time of next movement by the RNAP is drawn using this exponential distribution and recorded as *T*_*i*_ into the triple *P*_*i*_. That is, from Eq (7), the variable *T*_*i*_ is assigned by sampling this exponential distribution using the current value of *V*(*τ*_*i*_).

The likelihood of *P*_*i*_ entering a pause depends on the pause frequency under the calculated torque, *F*(*τ*_*i*_), and once this quantity is calculated, a uniformly distributed random number, *u* ∈ [0, 1] is drawn. If *u* ≥ *F*(*τ*_*i*_), the RNAP continues the elongation process. However, if *u* < *F*(*τ*_*i*_), the RNAP enters a pause. When *P*_*i*_ enters a pause, then the pause duration, *D*(*τ*_*i*_), is computed using Eq (21). The velocity of the RNAP is then reset to *V*(*τ*_*i*_) = 1/*D*(*τ*_*i*_), and the time of the next movement, *T*_*i*_ is drawn based on this velocity in the same manner that was described in the preceding paragraph. Therefore the expected value of the time of the next elongation for *P*_*i*_ is 1/*V*(*τ*_*i*_) = *D*(*τ*_*i*_). Note here that this duration is updated when a neighboring RNAP translocates (because the value of *τ*_*i*_ changes when that motion occurs), therefore the time of next elongation for the paused RNAP *P*_*i*_ is recalculated, and the pause duration changes accordingly. The simulation scheme also monitors other pieces of information that are crucial for our analysis, in particular the furthest downstream nucleotide occupied by a paused RNAP (position) as well as both the start time and the end time of each pause are recorded for a posteriori analysis purposes.

#### Incorporation of collisions

The other event that can affect elongation is a collision event. As mentioned previously, a collision occurs when the trailing RNAP, *P*_*i*_ must cease active elongation because the next nucleotide is already occupied by the leading RNAP, *P*_*i* − 1_. Since RNAPs cannot overlap nucleotides, or “hop over” each other as seen in specific cases with ribosomes during translation [51–53], *P*_*i*_ will halt elongation until the next nucleotide is free. When a collision occurs, *V*(*τ*_*i*_) is set to zero and the time of next movement is not drawn until *P*_*i*_ is free to move. Essentially, elongation of *P*_*i*_ is put on hold. This ensures that an elongation event for *P*_*i*_, when it is directly behind *P*_*i* − 1_, will not be chosen for execution; therefore no polymerases will overlap. When *P*_*i* − 1_ translocates, the rate of *P*_*i*_ is subsequently updated, and the next time of elongation for *P*_*i*_ is drawn. We consider the end of the collision to be when *P*_*i*_ translocates, as opposed to when the next nucleotide becomes available. The position, start time and end time of each collision is also recorded for later use in our analysis.

**Fig 11:**
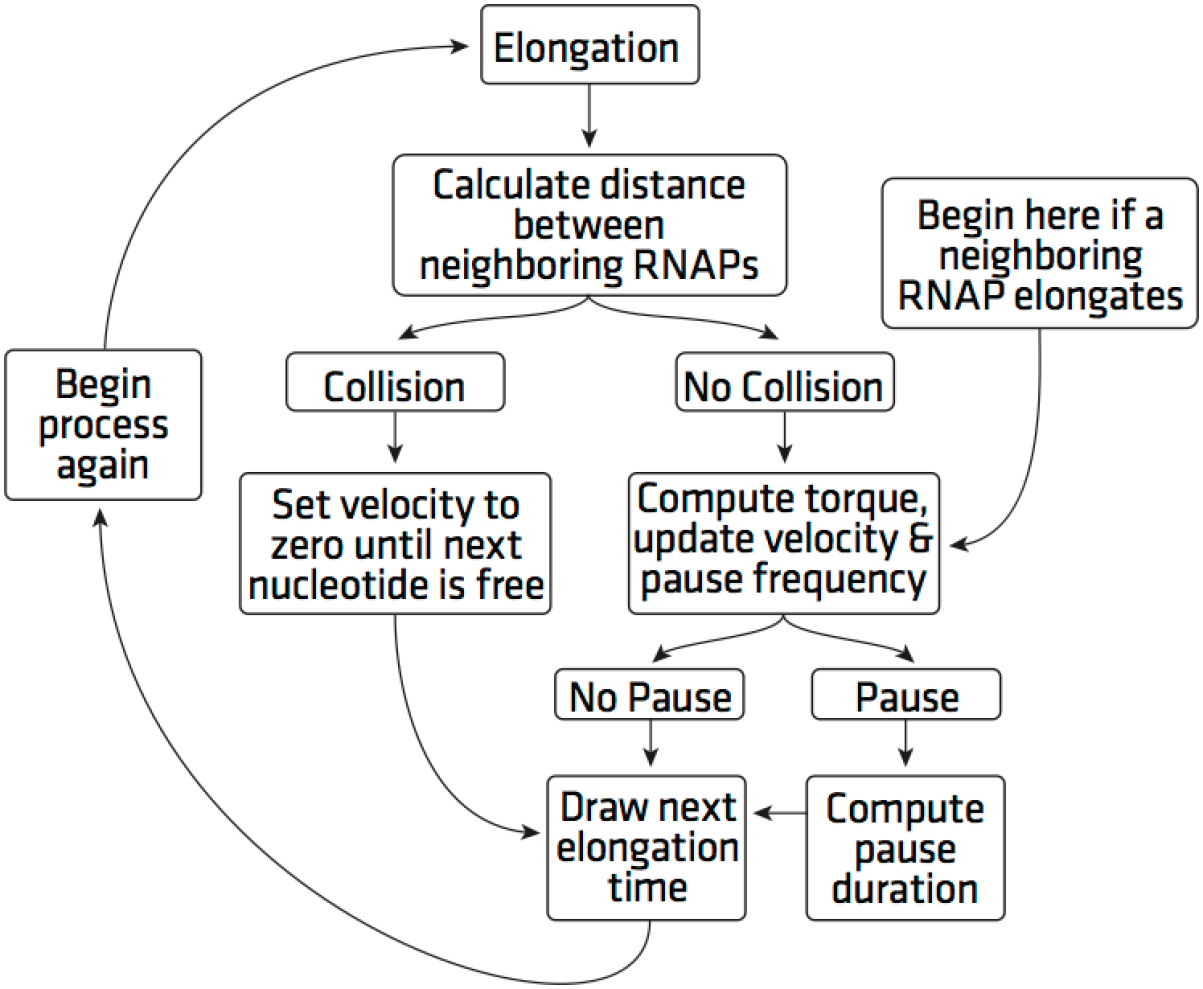
A flowchart for our simulation of the elongation process. RNAPs that translocate follow the chart from elongation and continue in order depending on if that RNAP experiences a collision or a pause. If a neighboring RNAP translocates, updates for torque, velocity, and pause frequency are calculated for the affected RNAPs starting with that block of the flowchart.

To summarize, upon elongation of *P*_*i*_, the process given in Algorithm 1 is performed for *P*_*i*_ and the neighboring RNAPs, *P*_*i* − 1_ and *P*_*i*+1_. A flowchart detailing the elongation process can be seen in Fig 11. The following subsections describe how we can simplify the model to recover the standard TASEP model. We then use this TASEP model for comparison with the ETAM model. A discussion of the parameters used for the numerical results is also presented below.

**Algorithm 1** This algorithm is performed after the elongation of *P*_*i*_.

1: *L*_*i*_, *L*_*i*+1_ ← using *n*_*i* − 1_, *n*_*i*_, *n*_*i*+1_

2: **if** *L*_*i*_ = 0 **then**

3:  *V*(*τ*_*i*_) = 0

4: *τ*_*i*_ ← using *L*_*i*_, *L*_*i*+1_, and *L*_0,*i*_, *L*_0,*i*+1_

5: *V*(*τ*_*i*_), *F*(*τ*_*i*_)←using *τ*_*i*_

6: *u* ← random(0, 1)

7: **if** *u* < *F*(*τ*_*i*_) **then** Pause

8:  *D*(*τ*_*i*_)←using *τ*_*i*_

9:  *V*(*τ*_*i*_) = 1/*D*(*τ*_*i*_)

10: **else** *u* ≥ *F*(*τ*_*i*_) No Pause, Do not update *V*

11: *T*_*i*_ ← *V*(*τ*_*i*_)*exp*(−*V*(*τ*_*i*_)*t*)

**Remark 1** *In order to calculate the transcription time of each RNAP, the time of initiation and termination is recorded. If an RNAP initiates but does not complete termination within the simulation time, then the initiation time is not saved, and only the transcription times for the RNAPs that terminate during the time of simulation are used for the subsequent analysis. While all pauses and collisions that occur are recorded, for the analysis presented here we only include the information from pauses and collisions that occur before the final recorded termination time during the simulation.*

#### Comparison to TASEP

In order to isolate the effect of the torque on the RNAP elongation, we compare the ETAM model to the TASEP model, which has the same stochastic structure as ETAM, with the key exception that the torque effects are not included. In particular, we use the same flowchart as in Fig 11 but the velocity, pause frequency, and pause duration are all constant values that correspond to Eqs (19), (20) and (21) with *τ* = 0. This is equivalent to RNAPs experiencing zero torque for the entire simulation. We use a constant velocity of *β* = *V*(0) which also gives a constant initiation rate of *α* ⋅ *β*. Since the RNAPs are elongating with a constant rate of *β* and we have boundary conditions corresponding to initiation with rate *α* ⋅ *β* and termination with rate *γ* ⋅ *β*, this simulation describes the typical TASEP simulation described previously.

Pauses are simulated similarly to the pauses in ETAM. Since the torque value is always zero, the velocity and pause frequency are *V*(0) and *F*(0) respectively. These values are held constant throughout the simulation. As described before, upon elongation a uniformly distributed random number, *u*, is drawn. If *u* < *F*(0) the RNAP will experience a pause. The duration of the pause *D*(0) is employed to recalculate the velocity as 1/*D*(0). Upon completion of the pause, the velocity is reset to *V*(0), and the time of the next elongation calculated and stored.

#### Simulation parameters

The two most important parameters governing the simulation events are the average elongation rate, denoted by *β* and the scalar that affects the rate of initiation, denoted by *α*. In the literature, *β* can vary from about 20 nt/s [24] up to about 90 nt/s [16] depending on several factors including the gene the RNAP is transcribing, environmental conditions, and the specific structure of the gene [19, 20]. The average elongation rate for RNAPs on an rrn operon gene has been shown experimentally to be approximately 90 nt/s [12, 18–20]. Note that this is different from the data calculated by Ma et. al. [24] where they measured the velocity for an RNAP under no torque at approximately 22 bp/s. This measurement is consistent with single-molecule experiments on certain E. coli gene sequences [54, 55]. In order to simulate transcription on an rrn operon, we multiply the velocity function described by Eq (19) by an appropriate scaling factor, *K*. The value of *K* is chosen so that an RNAP under no torque travels at 90 nt/s, which gives *K* ⋅ *V*(0) = *β* = 90 nt/s. Therefore, when drawing the next time for elongation of an RNAP, we use the exponential distribution *KV*(*τ*)*e*^−*KV*(*τ*)*t*^, with the expected value (*KV*(*τ*))^−1^.

While mathematically, the initiation parameter *α* can be as high as 1, in reality the values of *α* are much less than 1. Simulations were conducted using a variety of initiation rates ranging from *α* = 0.0001, which corresponds to an initiation every 109 seconds on average, to *α* = 0.0115, which corresponds to an initiation every 1.6 seconds on average. According to the literature, an rrn operon has approximately 31% coverage of the DNA strand by RNAPs [16]. Accounting for this percentage of coverage on a strand of DNA that is 5450 nts long occupied by RNAPs, each of length 35 nts, corresponds to approximately 50 RNAPs on the strand at any given time. Hence, the choice of the parameter range *α* ∈ [0.0001, 0.0115] is meant to mimic conditions ranging from light coverage of the DNA strand to a density of RNAPs that is beyond the experimentally observed conditions described above.

### ETAM Model Results from the Piecewise Linear Data Fit

The small number of data points given in [24] require us to extrapolate in order to characterize the relationships between the torque and the various physical quantities (elongation velocity, pause frequency and pause duration) for the ETAM model in the extreme cases where the torque values are near the melting or the stall cases, that is, where the absolute value of the torque is large. For those two cases, the functional relationships are constructed based on experimental biological data from [24] as well as other literature. The results presented in the Results Section at the beginning of this paper focused on that combination of information to inform the mathematical model; however, the choices made for the functional relationships were still somewhat arbitrary. The results of this section illustrate that the choices made for the case of torque values near either melting or stall are very crucial to the results of the mathematical model. The most important result shows that the cooperative effect discussed previously is observed for some choices of data fit constructions but not for others. Hence, the results reported in this section demonstrate the need for more experimental data over a larger range of torque values in order to produce a realistic mathematical model.

**Fig 12:**
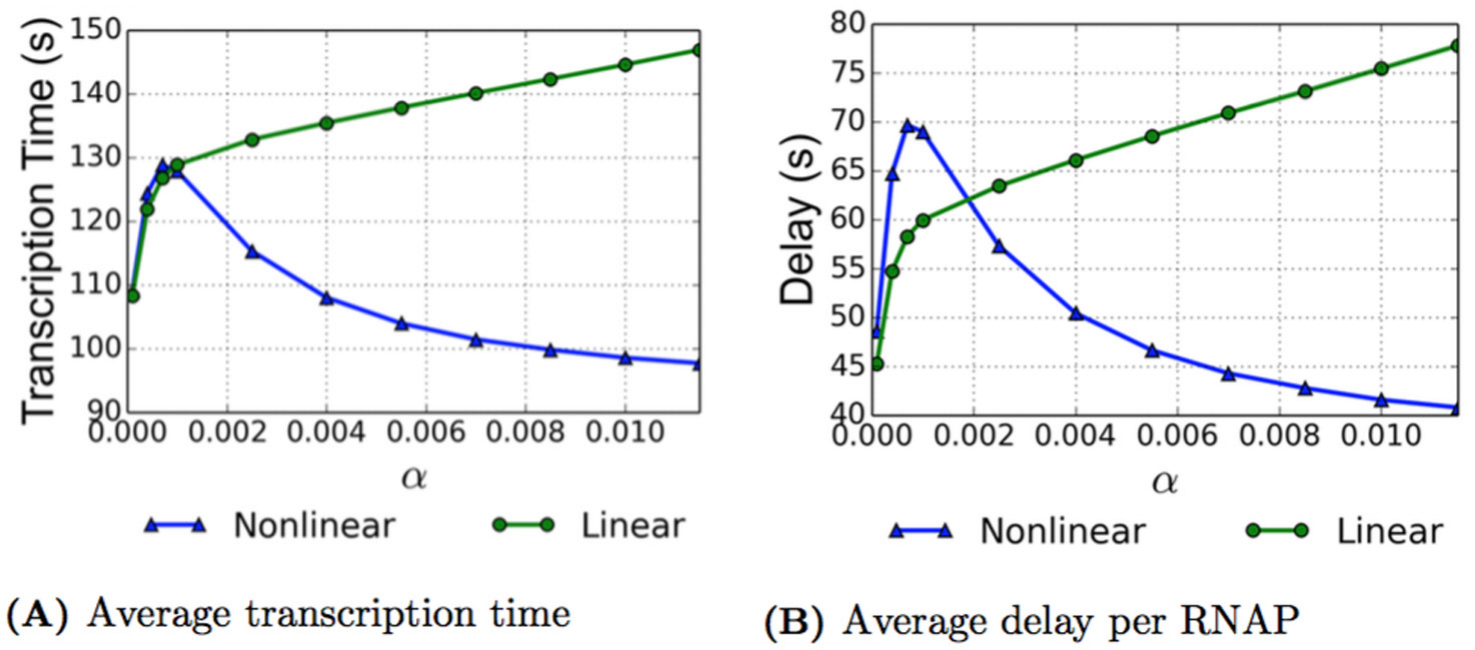
For the range of initiation rates *α* ⋅ *β*, we present our results for average transcription time (A) and average total delay (B) per RNAP in the ETAM model. The nonlinear model (blue triangles) and piecewise linear model (green dots), detailed in the sections Incorporating Experimental Data into the Model and Linear Fit to the Data respectively, are both plotted for comparison.

Although the average transcription time and total delay functions displayed for the ETAM model in the Results Section are largely concave up, the results are very different when using the piecewise linear curve fit discussed in the section Linear Fit to the Data for ETAM simulations. With the piecewise linear fit, the average transcription time and total delay have the same behavior as the nonlinear fit for small values of *α*, but the results continue to increase and remain concave down for the larger initiation rates. This is in sharp contrast to the behavior of the data generated using the nonlinear fit in the case of larger initiation rates. This can be seen in Fig 12.

For *α* = 0.0001 the average transcription time is approximately 108 seconds with a total delay of 45 seconds. This increases over the range of *α*, to a final average transcription time of 147 seconds and a delay of 78 seconds. This is a 36% increase in transcription times and a 73% increase in total delay. If we compare these to the values calculated for its nonlinear counterpart, we see that for the largest value of *α* = 0.0115, there is a 50% increase in transcription time and a 91% increase in delay for the piecewise linear fit. While the result with the piecewise linear fit is much closer to a 60 second average transcription time than TASEP, the cooperative effect of the nonlinear fit from the Results Section diminished for this particular choice of piecewise linear fit. The cooperative effect of the ETAM in the Results Section shows a decrease in transcription time as the coverage of the DNA strand increases to biologically relevant situations, and that effect is different than both the piecewise linear fit for ETAM and the TASEP case where transcription time monotonically increases as the coverage of the DNA strand increases.

### The Importance of the Nonlinear Pause Duration

**Fig 13:**
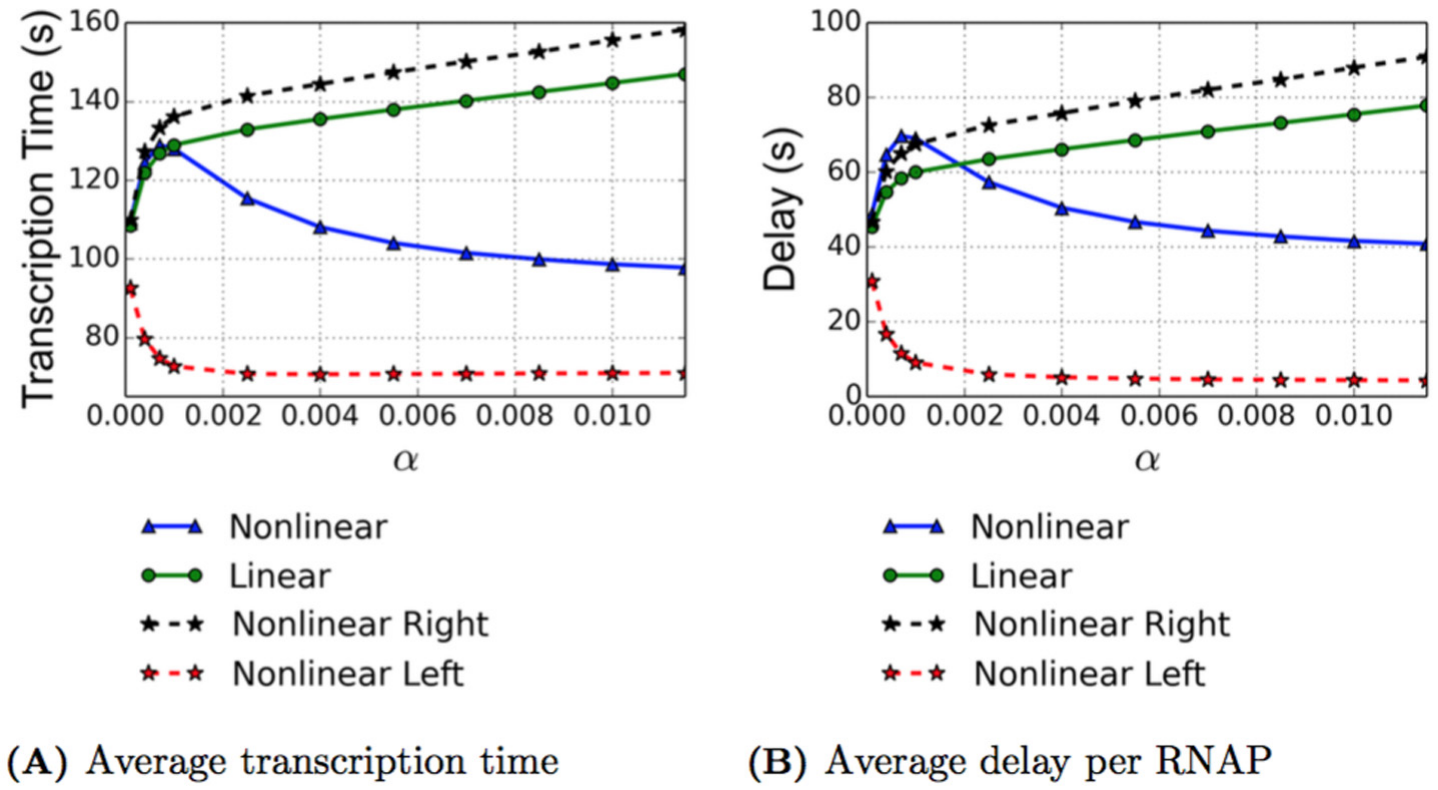
In addition to the nonlinear fit (blue triangles) and the piecewise linear fit (green dots), we also present our results for average transcription time (A) and average total delay (B) per RANP for a piecewise linear fit except for the pause duration on the interval [-10, 5], “Nonlinear Left” (red stars), or on the interval [5, 10], “Nonlinear Right” (black stars).

Because of the difference in results between the nonlinear fit of the data and that of a piecewise linear fit, we investigate which function or combination of functions drives this difference. Below we explore several possible combinations of piecewise linear and nonlinear data fit choices, and we find that the behavior is being driven by the pause duration function, specifically the pause duration for very low torque values near the melting threshold, see Fig 13. This figure compares the ETAM transcription time and delay results using the nonlinear fit for the pause duration function (Fig 9), and the piecewise linear fit for the pause duration function (Fig 10) with two other curves. The other curves show results when the pause duration function is constructed in a piecewise manner by combining portions of the piecewise linear and nonlinear fits in order to define other composite piecewise functions for the pause duration function. The curve labeled “Nonlinear Left” shows the results for average transcription time and average delay where the piecewise linear fit is used for all functions except the pause duration. The pause duration, instead, is a new piecewise defined function that is a combination of the nonlinear fit for *α* ∈ [-10, 5] and the piecewise linear fit for *α* ∈ [5, 11]. Similarly the curve labeled “Nonlinear Right” represents the results obtained using the nonlinear fit for *α* ∈ [5, 11] and the piecewise linear fit for *α* ∈ [-10, 5] for the pause duration function. As one can observe, the results for the piecewise linear fit and the nonlinear right fit are qualitatively similar. The most surprising are the results for the nonlinear left fit. For this curve fit, with large values of *α*, the RNAPs experience nearly 60 second transcription times with virtually no delay. The reason for this difference in results can be seen as we investigate the graph of pause duration for torque values near -10 pN⋅nm.

**Fig 14:**
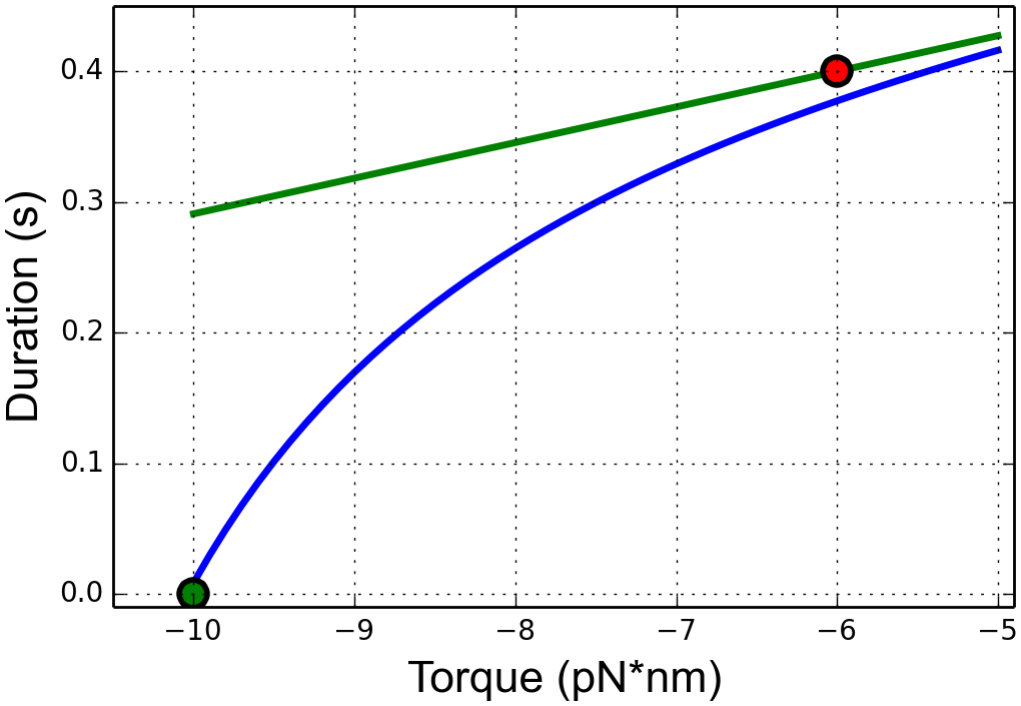
The differences in the pause duration for the piecewise linear fit (green) and the nonlinear fit (blue) at low torque values is highlighted here. This difference accounts for the very different results in average transcription time and average total delay depicted in Fig 12.

Fig 14 depicts the nonlinear pause duration and the piecewise linear pause duration for torque values in the interval [-10, -5]; it is essentially a “zoom in” of the graph in Fig 10C. It’s important to note that pause durations are not fixed but are calculated upon entering a pause and then recalculated when neighboring RNAPs elongate. However, when an RNAP is paused, the recalculated pause duration will always be smaller than the original because it is either being pulled from in front or pushed from behind by the elongation of a neighbor. Therefore even if the original pause duration assigned to the RNAP is large, the recalculated pause duration is likely to be small, as evidenced by the extremely small average pause durations in high coverage environments. Hence, the range of pause duration values for the case of small torque is very important. In the case of the nonlinear data fit with low torque values, the pause duration can be set as low as zero, in which case, the RNAP is released from the pause state and is free to elongate. However for the case of the piecewise linear data fit, the lowest pause duration possible is approximately 0.3 seconds. With the large amount of pauses experienced by an RNAP, the difference in pause duration for low torque values is driving the difference in behavior between the nonlinear and piecewise linear data fit.

To finish our discussion on pause duration we again consider Fig 13. We concentrate on the difference in the results of the “Nonlinear” data fit and the “Nonlinear Left” data fit whose pause duration is linear for *α* ∈ [5, 10] and nonlinear for *α* ∈ [-10, 5]. If the behavior of the results is driven by the pause duration for low torque values, how do we account for this difference, as they have the same pause duration for those torque values? This difference can be attributed to the pause frequency function. Recall, the “Nonlinear Left” data fit has the piecewise linear fit for velocity and pause frequency. The piecewise linear fit for large torque values would give a pause frequency of 0.049 when the torque is 11 pN⋅nm, as opposed to a pause frequency of 1 for the nonlinear fit. The RNAPs will experience significantly fewer pauses with the linear pause frequency, encountering on average 146 pauses when *α* = 0.0115, as opposed to 2169 pauses. Regardless of how short the duration, a very large number of these minor interruptions in elongation (corresponding to a large value of the frequency function) can have a large effect on the overall transcription time of an RNAP. With this in mind, we investigate how the value of the pause frequency function when torque is 11 pN⋅nm can affect the results of the model.

### Results from Different Pause Frequency Functions

**Fig 15:**
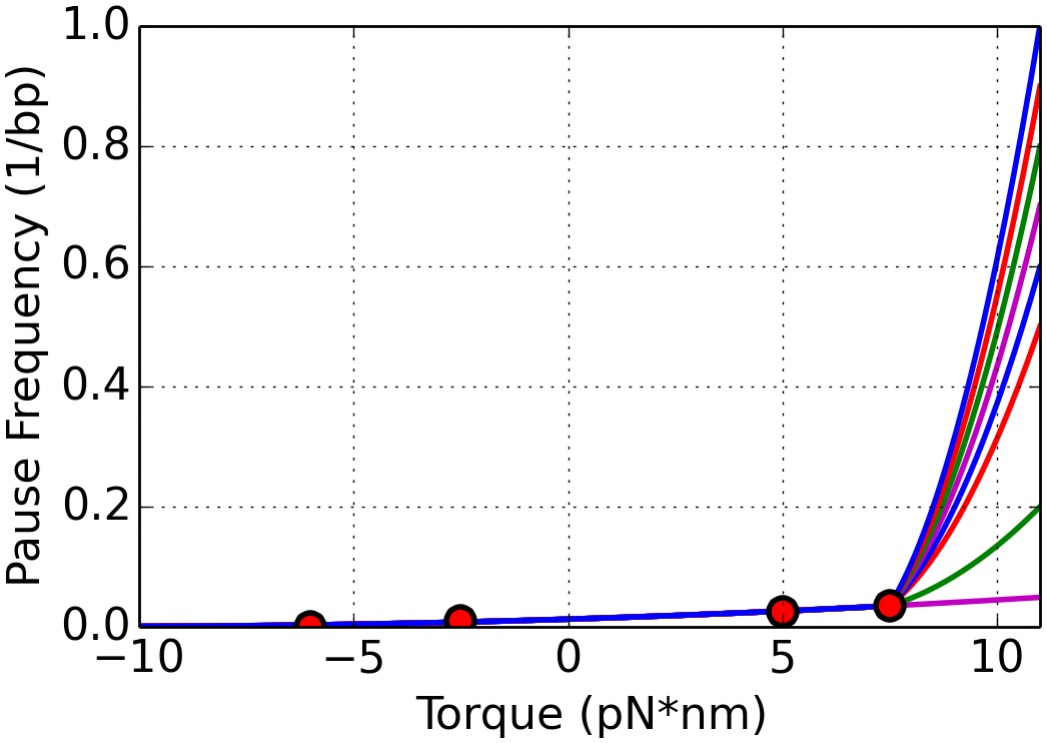
Pause frequency function where the value at the end point, 11 pN⋅nm is raised. The data (red dots) is fit with one quadratic function up to 7.5 pN⋅nm. A second quadratic function is used to fit the end point, with the exception of the lowest function which is the original quadratic for the entire interval.

**Fig 16:**
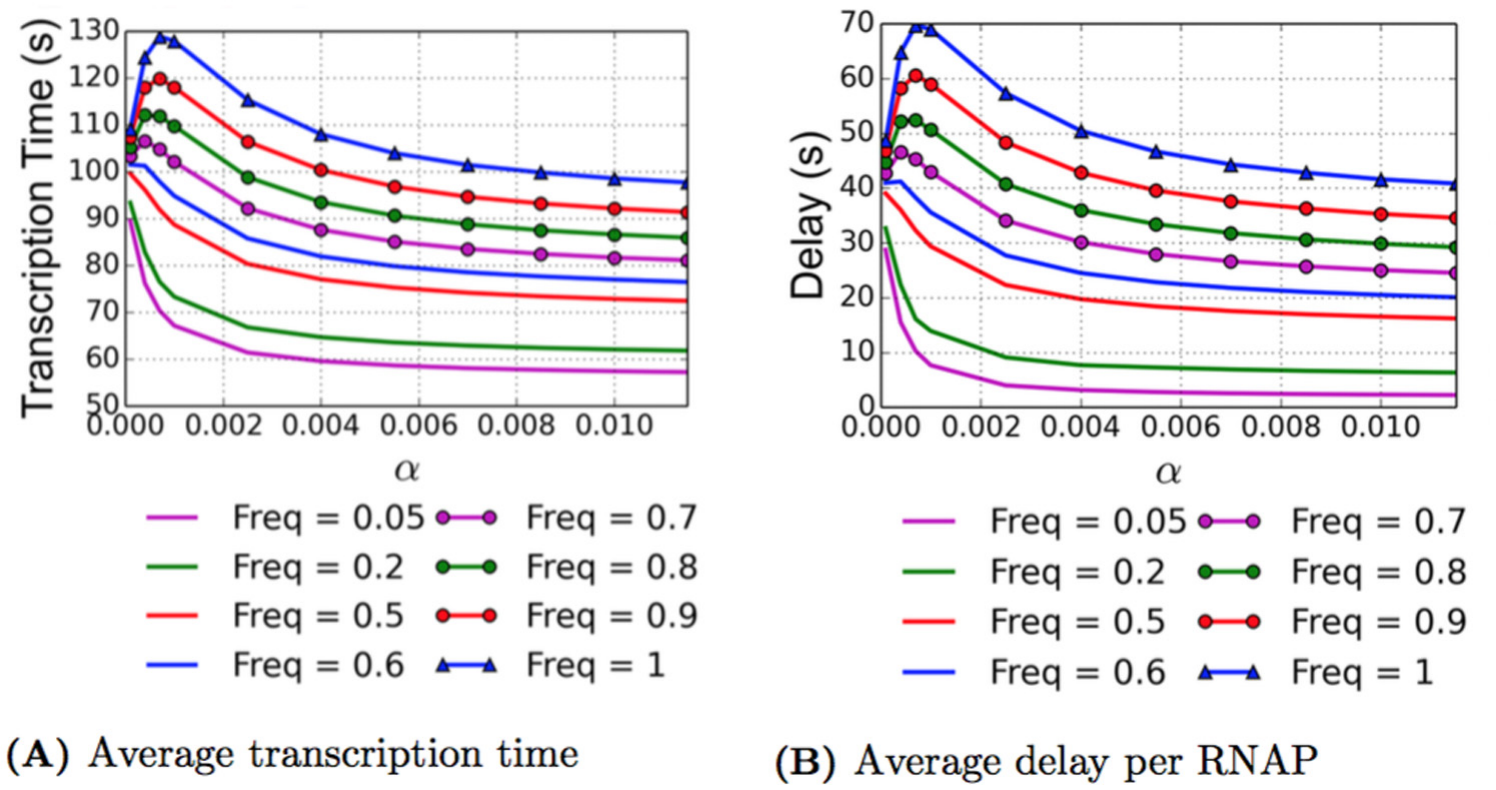
For the range of initiation rates *α* ⋅ *β*, these results show average transcription time (A) and average total delay (B) over different fits of the pause frequency function. The legend labels the pause frequency at the highest torque value.

As mentioned earlier, the choices for data fit in the cases of very high torque values and very low torque values were somewhat arbitrary with the limited data points. One choice which has proven to be crucial is to use a pause frequency of 1 when torque is 11 pN⋅nm, the approximate value for stall torque. Here we illustrate the impact of that choice on our results. Using the nonlinear data fit for pause duration and velocity, as in Eqs (21) and (19), we perform a set of calculations for various choices for the function value when torque is 11 pN⋅nm. We continue to fit the pause frequency function using quadratic functions similar to Eq (20); however the value of the pause frequency function when the torque value is 11 pN⋅nm is allowed to range over a variety of values smaller than 1 in order to compare the results, see Fig 15. The values for pause frequency used when torque is 11 pN⋅nm are {0.2, 0.5, 0.6, 0.7, 0.8, 0.9}. We also report the results for the original pause frequency function that takes on the value of 1 at the stall torque. Also included are the results for the case where the pause frequency function data is fit with one quadratic function which has a value of approximately 0.05 at stall torque. The average transcription time and total delay for all of these different choices for data fit can be seen in Fig 16A and 16B respectively. Pause frequency increasing to 0.05 and 0.2 at stall torque have the fastest transcription times, both being very close to a 60 second transcription time and, in the case of the lowest pause frequency, actually being faster than a 60 second transcription time. These two pause frequency functions give results that agree extremely well with experimental data for large values of *α*. If we consider the difference in the average number of pauses per RNAP under these different pause frequency functions, we can understand the difference in delay. Table 4 shows the average number of pauses per RNAP, average pause duration, and the corresponding delay due to pauses when using the various pause frequencies for the value of *α* = 0.0115, as shown in Fig 16. By examining the delay values in Table 4 and comparing these to Fig 16B, one can see that the pause delay contributes the majority of the time toward the total delay each polymerase experiences. The pause delay is influenced mostly by the number of pauses per polymerase which is a direct result of the choice of the pause frequency function.

**Table 4:** The average number of pauses per RNAP, average pause duration, and average pause delay per RNAP due to pauses experienced per RNAP for the different pause frequency functions. Frequency represents the value of the pause frequency function when torque is 11 pN⋅nm. All of the data reported is for *α* = 0.0115.

Frequency	# of Pauses	Duration (s)	Delay (s)
0.05	162	0.013	2.106
0.2	433	0.014	6.062
0.5	1013	0.016	16.208
0.6	1219	0.016	19.504
0.7	1443	0.017	24.531
0.8	1668	0.017	28.356
0.9	1909	0.018	34.362
1	2169	0.018	39.042

Another interesting result is the shift in behavior from frequency 0.5 to frequency 0.6. The results for lower frequency values are concave up over the range of *α*. However with pause frequency up to 0.6 we begin to see a global maximum when *α* = 0.0004. For pause frequency greater than or equal to 0.8 the global maximum is when *α* = 0.0007. For values of *α* larger than 0.0007, the coverage of the DNA strand is large enough that the RNAPs feel a substantial effect from their neighboring polymerases and begin to experience a cooperative effect. It is in this range that while the polymerases experience more pauses, the decrease in the pause duration is enough to shorten the total delay. We believe this is the range of initiation values where the RNAPs are now close enough for the torsional interaction to push a paused RNAP back into elongation, as proposed by Epshtein et. al. [17], substantially quicker than they had been previously. As the values of *α* increase beyond 0.0007, this cooperation becomes even more pronounced. Regardless of pause frequency, the overall cooperative behavior is clear from the decrease in delay and transcription times for the larger coverages.

Mathematically, choosing a value for pause frequency equal to 1, or even close to 1, when torque is 11 pN⋅nm is the natural choice as this is the stall torque. However, if one is attempting to fit the experimental data with one function, one would choose a pause frequency close to 0.2. For that case, we have 30% coverage of the DNA strand with an average transcription time of just under 62 seconds at the highest initiation rate. This agrees very well with the results presented by Neuman et. al. [4]. In order to properly tune the ETAM model proposed here, more data is necessary for the extreme cases near the stall torque and melting torque.

**Fig 17:**
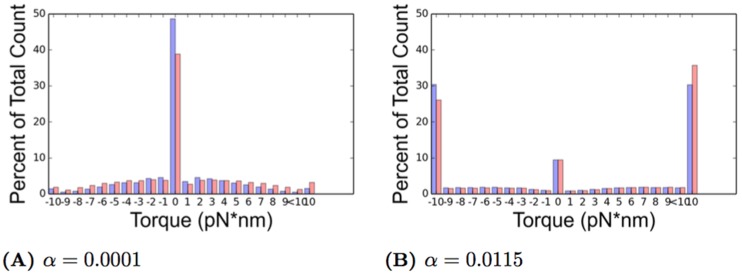
The percentage of times a torque value is calculated in a baseline (blue) simulation and a pause (red) simulation is presented here as a histogram. The histogram bars at -10 and 10 represent the percentage of the total number of torque measurements within the simulation that those exteme values are computed. Each of the other bars represents the percentage total measurements that lie within (-10, -9], (-9, -8], etc., with the exception of the bars at the label <10. The histogram bars at <10 represent the percentage of torque values computed in (9, 10). (A) shows the torque values measured for *α* = 0.0001 which corresponds to the lowest initiation rate simulated, while (B) shows the torque values measured for *α* = 0.0115 which corresponds to the highest initiation rate simulated.

In order to illustrate the importance of accurate measurements near the stall torque and the melting torque for the ETAM model, we investigate how often these torque values are sampled during the simulations of the ETAM model over the range of *α*. Fig 17 depicts the number of times each torque value is calculated in a simulation as a percentage of the total number of torque values that are computed, displayed as a histogram. We show the results for both the baseline simulation (no pauses), and the pause simulation for *α* = 0.0001 (Fig 17A) and *α* = 0.0115 (Fig 17B). In these simulations, we count the number of times the torque values of 10 pN⋅nm and -10 pN⋅nm are computed, and we plot this percentage as histogram bars at -10 and 10 respectively. In between these values we tabulated the number of times a torque value fell in the interval (-10, -9] and plotted that percentage in the histogram bars at -9. The percentage of torque values in (-9, -8] were plotted in the histogram bars at -8, and so on, up to the torque value of 9. The percentage of values in the interval (9, 10) were plotted under the label of <10. As shown in Fig 17, for our lowest initiation rate (*α* = 0.0001) the algorithm computes a torque of 0 pN⋅nm approximately 50 percent of the time in the baseline simulation and just under 40 percent for the pause simulation. This is to be expected as many of the RNAPs for this value of *α* are transcribing as single molecules and therefore would not generate torque values away from 0 pN⋅nm. For *α* = 0.0115, the results are very different. The interaction between RNAPs causes the 0 pN⋅nm to be computed only 10% of the time. The extreme values of -10 pN⋅nm and 10 pN⋅nm are calculated the most often. In the pause simulation -10 pN⋅nm is computed approximately 25% of the time and 10 pN⋅nm about 35% of the time. This high percentage is due to the fact that the RNAPs are very close together at this high density and therefore the interactions between them are extremely strong. As a result, the torque values related to the extreme cases of stall and melting along with the choices one uses to construct the functional relationships between torque and all three of velocity, pause duration, and pause frequency at these extreme torque values combine to create a large impact on the ETAM results.
